# Quantum-enhanced radiometry via approximate quantum error correction

**DOI:** 10.1038/s41467-022-30410-8

**Published:** 2022-06-09

**Authors:** W. Wang, Z.-J. Chen, X. Liu, W. Cai, Y. Ma, X. Mu, X. Pan, Z. Hua, L. Hu, Y. Xu, H. Wang, Y. P. Song, X.-B. Zou, C.-L. Zou, L. Sun

**Affiliations:** 1 Center for Quantum Information, Institute for Interdisciplinary Information Sciences, Tsinghua University, Beijing, 100084 P. R. China; 2 Key Laboratory of Quantum Information, CAS, University of Science and Technology of China, Hefei, Anhui 230026 P. R. China

**Keywords:** Quantum metrology, Quantum information, Qubits, Quantum optics

## Abstract

Quantum sensing based on exotic quantum states is appealing for practical metrology applications and fundamental studies. However, these quantum states are vulnerable to noise and the resulting quantum enhancement is weakened in practice. Here, we experimentally demonstrate a quantum-enhanced sensing scheme with a bosonic probe, by exploring the large Hilbert space of the bosonic mode and developing both the approximate quantum error correction and the quantum jump tracking approaches. In a practical radiometry scenario, we attain a 5.3 dB enhancement of sensitivity, which reaches 9.1 × 10^−4^ Hz^−1/2^ when measuring the excitation population of a receiver mode. Our results demonstrate the potential of quantum sensing with near-term quantum technologies, not only shedding new light on the quantum advantage of sensing, but also stimulating further efforts on bosonic quantum technologies.

## Abstract (web summary)

Exotic quantum states can be advantageous for sensing, but are very fragile, so that some form of quantum error correction is needed. Here, the authors show how approximate QEC helps overcoming decoherence due to noise when measuring the excitation population of a receiver mode in a superconducting circuit.

## Introduction

The large Hilbert space of a quantum system and the quantum superposition principle offer the potential advantages of quantum physics in many applications and laid the foundation of quantum information science^1^. Recently, supported by the established quantum state engineering and control techniques, quantum sensing emerges as one of the most promising near-term applications to achieve quantum advantage and has attracted tremendous attentions^2–6^. For a sensor interrogation Hamiltonian *H*_int_, the intriguing Greenberger–Horne–Zeilinger entanglement states of a collection of spins^7^ or the Schrödinger-cat-like states in a large Hilbert space^8–11^ could enhance the sensitivity of a quantum probe, because they provide a large variance of energy $\left\langle {H}_{{{{{{{{\rm{int}}}}}}}}}^{2}\right\rangle -{\left\langle {H}_{{{{{{{{\rm{int}}}}}}}}}\right\rangle }^{2}\propto {N}^{2}$ with *N* being the number of excitations. However, these exotic quantum states are also more prone to environmental noises, and thus the coherence times are reduced and the ultimate sensing sensitivity is hard to be enhanced^12–14^.

Fortunately, the large Hilbert space also offers the redundancy for realizing quantum error correction (QEC) that protects quantum states from decoherences and imperfections. It has been expected that the Heisenberg limit, i.e., the sensitivity of the sensing scales with the measurement time (*T*) and excitation number as $\propto {\left(NT\right)}^{-1}$, could be achieved by protecting the exotic quantum states via QEC^15–21^, in sharp contrast to the standard quantum limit $\propto {\left(NT\right)}^{-1/2}$. However, these QEC-based quantum sensing schemes demand stringent conditions in experiments. On one hand, the non-local QEC operations are challenging for multi-qubit systems^22–24^. Although a pioneering experiment proves the principle of QEC-enhanced sensing by prolonging the coherence time of a single electron spin^25^, the extension to a larger Hilbert space is absent. On the other hand, the theoretical assumptions of perfect ancilla or error-free quantum operations are impractical, and the noises of experimental systems could not meet the orthogonality requirement in general^21^. The unraveled fact that the Heisenberg limit could not be practically attainable^26,27^ discourages further experimental exploration of quantum-enhanced sensing via QEC.

In this work, we demonstrate the enhancement of the sensing sensitivity by approximate QEC with a bosonic probe. Instead of pursuing the Heisenberg limit, our quantum sensing is implemented with optimized experimental strategies in a hardware-efficient superconducting architecture. By using non-exact QEC codes based on two-component Fock states for carrying the coherence, the sensing information could be protected by QEC, and the imperfection due to decoherence could even be further suppressed by mitigating the ambiguity of the quantum evolution trajectories. Benefiting from both the enhanced sensing-information-gain rate and prolonged coherence time of the exotic quantum states, the bosonic probe is applied for practical radiometry and achieves a detection limit of the receiver excitation population of 9.1 × 10^−4^ Hz^−1/2^, which shows a 5.3 dB enhancement of the sensitivity by QEC compared to the encoding with the two lowest Fock states. Our work develops practical quantum sensing technologies, proves the quantum enhancement, and could also stimulate further experimental efforts in this direction.

## Results

### Theory of approximate bosonic QEC for sensing

**Fig. 1:**
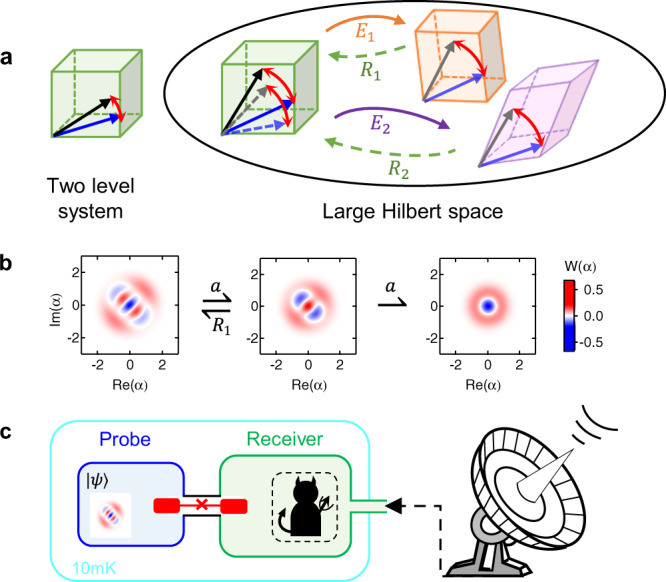
Schematic of practical quantum-enhanced sensing. **a** The principle of quantum sensing with approximate quantum error correction (QEC). The errors $\left\{{E}_{1},{E}_{2},...\right\}$ map the quantum states in the code space to disjoint subspaces and the recovery operations $\left\{{R}_{1},{R}_{2},...\right\}$ covert the states back to the code space with the acquired phase being preserved. However, the recovered state in the code space is deformed due to the nature of the approximate QEC, as illustrated by the dashed arrows. **b** One example of practical quantum sensing protocol with a bosonic probe. The Wigner functions illustrate the evolution of the probe quantum state: When the probe state is initialized to $(\left|1\right\rangle +i\left|3\right\rangle )/\sqrt{2}$, the phase can be preserved even if there is a single-photon-loss error (*a*), which could be tracked and corrected via the recovery operation (*R*_1_). **c** Schematic of the experimental setup for the quantum radiometry implemented with a superconducting architecture. The device is constructed with a bosonic probe that couples to a receiver mode.

Figure 1 illustrates the principle of quantum-enhanced sensing. When a single two-level system (TLS) is used to probe an external field through coherent coupling, the quantum state in the two-dimensional Hilbert space would acquire a phase proportional to the sensing duration *t*_int_. By contrast, when extending the probe to a higher dimension, it is possible to find a subspace in which the phase accumulation rate is higher, while the rotation angle can be preserved even though the states are mapped to disjoint error subspaces by noises. Therefore, the sensitivity could be enhanced by both the larger rate and the QEC protection. A single bosonic mode provides an excellent probe for realizing such an idea in practice because of its large Hilbert space dimension and hardware-efficient quantum control capability^10,11,28^. In general, a bosonic probe could sense a physical quantity *ω* through the interrogation Hamiltonian

1 $${H}_{{{{{{{{\rm{int}}}}}}}}}/\hslash =\omega {a}^{{{{\dagger}}} }a,$$

where *a* denotes the annihilation operator of the probe and *ℏ* is the Planck constant.

However, exact QEC codes for sensing with such a model are excluded, since the photon loss errors due to damping are not orthogonal to *H*_int_ [see Supplementary Note 1]. Because of the same reason, the Heisenberg limit could not be achievable in principle with such a sensing scheme. Nevertheless, the protection of the probe from noise by an approximate QEC can still be advantageous in practical metrology applications as demonstrated below. For the convenience of experiments, we propose the two-component Fock state subspace ${{{{{{{\rm{span}}}}}}}}\left\{\left|m\right\rangle ,\left|n\right\rangle \right\}$ (*m* < *n*) as the QEC code space for sensing. The evolution of the probe state after an interrogation time *t*_int_ becomes

2 $$|{\psi }_{m,n}\rangle ={\alpha }_{m,n}\left|m\right\rangle +{\beta }_{m,n}{e}^{-i(n-m)\omega {t}_{{{{{{{{\rm{int}}}}}}}}}+i{\varphi }_{0}}\left|n\right\rangle ,$$

where ${\alpha }_{m,n},{\beta }_{m,n}\in {\mathsf{R}}$ are the amplitudes and *φ*_0_ is the initial phase. It is easy to verify that for loss errors up to *m* photons, the acquired phase $\varphi =\left(n-m\right)\omega {t}_{{{{{{{{\rm{int}}}}}}}}}$ is preserved with the phase accumulation rate $\left(n-m\right)\omega$ being fixed irrespective of the time when the photon jump occurs. By repetitively implementing the recovery operations ${R}_{j}=\left|m\right\rangle \left\langle m-j\right|+\left|n\right\rangle \left\langle n-j\right|+{\widetilde{R}}_{j}$ for the *j*-photon-loss error, with ${\widetilde{R}}_{j}$ being a complementary operator to make *R*_*j*_ unitary, the phase coherence could be protected in the code space. Therefore, such a code in a high-dimensional Fock space could simultaneously enhance the phase accumulation rate by *n* − *m* times when compared to the encoding with the lowest two levels and prolong the coherence time via QEC. However, the coherence time could only be enhanced by a limited factor, since the code is an approximate QEC code, and the amplitudes of the probe state *α*_*n*,*m*_ and *β*_*n*,*m*_ change after each photon loss, as illustrated by the deformation of the error subspaces and the recovered state in the code space in Fig. 1a. This deformation will be one limitation of the approximate QEC and could be mitigated by tracking the photon loss errors as discussed below.

An example of this proposal is schematically shown in Fig. 1b with a probe state $|{\psi }_{1,3}\rangle =\frac{1}{\sqrt{2}}\left(\left|1\right\rangle +{e}^{i\pi /2}\left|3\right\rangle \right)$, i.e., *m* = 1 and *n* = 3. The single-photon-loss error maps the state to $\frac{1}{2}\left(\left|0\right\rangle +\sqrt{3}{e}^{i\pi /2}\left|2\right\rangle \right)$ and recovery *R*_1_ converts the state back to the code space, while the Wigner functions of both states have the same rotation angle and symmetry. However, the phase information is completely corrupted for a two-photon-loss error since *m* < 2.

### Experimental architecture and scheme

To demonstrate the efficacy of the approximate bosonic QEC scheme for sensing, we first experimentally characterize the performance of the scheme by measuring a virtual phase (*φ*_0_) introduced in the initial probe state instead of an acquired phase (*φ*). Our experimental device schematically shown in Fig. 1c consists of a superconducting transmon qubit as an ancilla dispersively coupled to two three-dimensional cavities^29–31^. The cavity (blue) with a long coherence time (*T*_1_ = 143 μs) serves as the probe, while the ancilla and the other short-lived cavity (green) assist in the manipulation and readout of the probe, respectively [see Methods for more details]. In the radiometry experiments studied later, the readout cavity serves as a receiver to collect microwave signals from outside, while the probe can sense the excitation in the receiver through the cross-Kerr interaction.

**Fig. 2:**
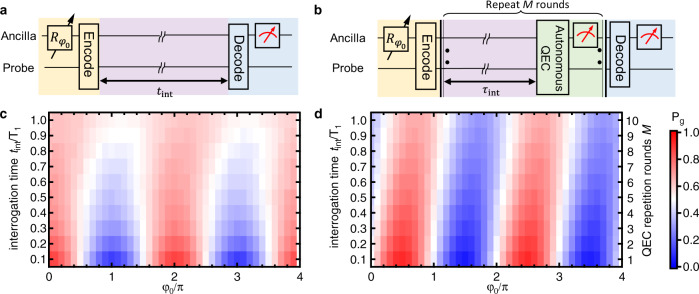
The sensing scheme with QEC. **a** Quantum circuit of the sensing scheme without QEC. After the encoding process, the probe evolves freely for an interrogation time *t*_int_, and is read out through detection of the ancilla following a decoding process. **b** Scheme for the QEC-enhanced sensing with the interrogation interleaved with QEC operations. The QEC operation consists of a QEC pulse and detection and a conditional reset of the ancilla. The interrogation time is *t*_int_ in **a** and *t*_int_ = *Mτ*_int_ in **b**, with *M* from 1 to 10 being the repetition number of QEC. **c** Measurement results of *P*_*g*_ without QEC for the probe state $|{\psi }_{1,3}\rangle =(\left|1\right\rangle +{e}^{i{\varphi }_{0}}\left|3\right\rangle )/\sqrt{2}$ against the initial phase *φ*_0_ and with *t*_int_ ranging from 0.1*T*_1_ to *T*_1_. **d** Performance of the QEC-enhanced sensing for the same probe state with an optimized interval *τ*_int_ = 0.1*T*_1_. The shift of fringes with the increasing *M* is mainly caused by the QEC-induced phase.

As shown by the experimental circuits in Fig. 2a, b, the ancilla assists the encoding and decoding of the probe by mapping the ground and the excited states $\{|g\rangle ,|e\rangle \}$ to the two Fock states $\left\{\left|m\right\rangle ,\left|n\right\rangle \right\}$. The sensing procedure is reminiscent of the Ramsey interferometer^32^ with the output probability of $|g\rangle$ as ${P}_{g}=A+B\cos (\varphi +{\varphi }_{0})$ manifesting the interference fringe, where *A* and *B* are the fitting parameters. Since the decay rate of Fock state $\left|m\right\rangle$ is proportional to the photon number *m*, we fix *m* = 1 and select *n* = 3, 5, 7 for a relatively small decay rate. The corresponding error set of the probe is $\{{E}_{0}={e}^{-{t}_{{{{{{{{\rm{int}}}}}}}}}{a}^{{{{\dagger}}} }a/2{T}_{1}},{E}_{1}=\sqrt{1-{e}^{-{t}_{{{{{{{{\rm{int}}}}}}}}}/{T}_{1}}}{e}^{-{t}_{{{{{{{{\rm{int}}}}}}}}}{a}^{{{{\dagger}}} }a/2{T}_{1}}a\}$ for zero- and single-photon-loss errors with an interrogation time *t*_int_, and we only tackle the dominant error *E*_1_ by *R*_1_. The QEC of the probe state is implemented through an autonomous manner^33,34^, i.e., by implementing a unitary operation on the joint state of the probe and the ancilla, regardless of whether *E*_1_ has occurred or not, to transfer the error entropy associated with the encoded probe state to the ancilla, followed by a reset of the ancilla [see Methods]. This autonomous QEC, therefore, does not require an error detection. Note that *α*_*m*,*n*_, *β*_*m*,*n*_, and *τ*_int_ are optimized in order to maximize the visibility of the output fringes for each $|{\psi }_{m,n}\rangle$ in the following experiments; therefore, the best detection sensitivity can be achieved experimentally [see Supplementary Note 2].

Figure 2c, d compares the measured probability *P*_*g*_ against the virtual phase *φ*_0_ and the sensing duration *t*_int_ for the cases without and with the protection by QEC. The results with QEC indeed show a much slower decaying of the fringe visibility. To evaluate the potentially achievable improvement of the sensing performance, we extract the Ramsey visibility against *φ*_0_ and derive the normalized quantum Fisher information (QFI) ${{{{{{{\mathcal{Q}}}}}}}}$ with respect to *t*_tot_, which is the total experimental time for a single-shot measurement including the initialization, encoding, interrogation, decoding, and readout, as well as the time needed for the QEC process and feedback when QEC is performed [see Methods]. ${{{{{{{\mathcal{Q}}}}}}}}$ determines the best achievable sensitivity of *ω* in a unit time, i.e.,

3 $${\sigma }_{\omega }\ge 1/\sqrt{{{{{{{{\mathcal{Q}}}}}}}}},$$

which is in the unit of ${{{{{{{\rm{Hz}}}}}}}}/\sqrt{{{{{{{{\rm{Hz}}}}}}}}}$ and corresponds to the noise floor of our sensor.

**Fig. 3:**
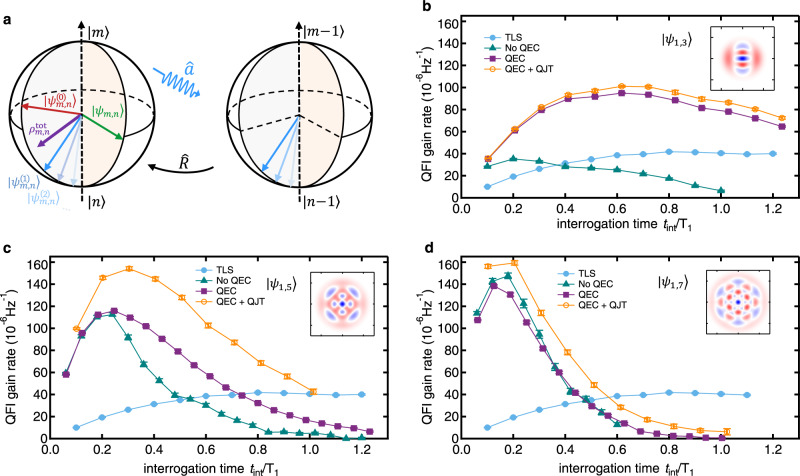
Normalized quantum Fisher information ${{{{{{{\mathcal{Q}}}}}}}}$. **a** The Bloch-sphere illustration of the quantum jump tracking (QJT). For a two-component Fock state $|{\psi }_{m,n}\rangle$, although it could be confined in the code space ${{{{{{{\rm{span}}}}}}}}\left\{|m\rangle ,|n\rangle \right\}$ via QEC, the amplitudes vary depending on the number (*j*) of the single-photon-loss errors occurring, i.e., $|{\psi }_{m,n}\rangle \mapsto |{\psi }_{m,n}^{(j)}\rangle$. The output would be a mixed state ${\rho }_{m,n}^{{{{{{{{\rm{(tot)}}}}}}}}}$, if different evolution trajectories could not be distinguished. **b**–**d** Quantitative performance of different quantum sensing strategies characterized by ${{{{{{{\mathcal{Q}}}}}}}}$ for the probe states $|{\psi }_{1,3}\rangle$, $|{\psi }_{1,5}\rangle$, and $|{\psi }_{1,7}\rangle$, respectively. TLS: the two-level system encoding with the two lowest Fock states. QEC and No QEC: results with and without QEC, respectively. QEC+QJT: the strategy that combines QEC and QJT. The error bars are obtained through error propagation of the fit parameter uncertainties. Inset: Wigner functions of the corresponding probe states, with the same axes and color scale bar as in Fig. 1b.

The results of ${{{{{{{\mathcal{Q}}}}}}}}$ with and without QEC are summarized in Fig. 3b–d, and ${{{{{{{\mathcal{Q}}}}}}}}$ of the simple TLS case with the probe being encoded in the lowest two levels is also provided as a reference. All curves of ${{{{{{{\mathcal{Q}}}}}}}}$ first grow up with *t*_int_, since a longer interrogation time gives a larger phase acquisition. However, due to more accumulated decoherence, ${{{{{{{\mathcal{Q}}}}}}}}$ saturates at an optimal *t*_int_ and decreases for even longer *t*_int_. Benefiting from the larger Hilbert space dimension, the best achievable ${{{{{{{\mathcal{Q}}}}}}}}$ without QEC increases with *n*, while the optimal *t*_int_ decreases with *n* due to the shorter Fock state lifetime. When performing QEC, ${{{{{{{\mathcal{Q}}}}}}}}$ is clearly improved than that without QEC especially when *t*_int_ exceeds the optimal value, confirming the enhanced coherence time by QEC. Comparing the results for different *n*, the improvement induced by QEC reduces with increasing *n*, due to the stronger uncorrectable noise effects for larger *n* and also higher operation errors when performing QEC. We note that the signal-induced dephasing error, however, could be negligible for current experimental parameters [see Supplementary Note 1].

### Quantum jump tracking (QJT) approach

As mentioned earlier, one main limitation on the performance of the approximate QEC would be the code state deformation, which causes decoherence even when the QEC is successfully implemented. For example, after one round of QEC, the amplitudes of $|{\psi }_{m,n}\rangle$ evolve as $\{{\alpha }_{m,n},{\beta }_{m,n}{e}^{-\left(n-m\right){\tau }_{{{{{{{{\rm{int}}}}}}}}}/2{T}_{1}+i{\varphi }_{0}}\}$ and $\{{\alpha }_{m,n},\sqrt{\frac{n}{m}}{\beta }_{m,n}{e}^{-\left(n-m\right){\tau }_{{{{{{{{\rm{int}}}}}}}}}/2{T}_{1}+i{\varphi }_{0}}\}$ (normalization factors are neglected) for *E*_0_ and *E*_1_ occurring, respectively, with the relative amplitude of the two Fock components being either amplified or suppressed. As illustrated in Fig. 3a, the final probe state ${\rho }_{m,n}^{{{{{{{{\rm{(tot)}}}}}}}}}$ becomes a mixed state composing of different possible quantum evolution trajectories ($|{\psi }_{m,n}^{(0)}\rangle$, $|{\psi }_{m,n}^{(1)}\rangle$, ...), although the phase is preserved irrespective of the total number of photon jumps during *τ*_int_. If we can distinguish the number of photon jumps that have occurred, the ambiguity of the possible trajectories could be mitigated, and thus ${{{{{{{\mathcal{Q}}}}}}}}$ could be improved.

Therefore, we propose and demonstrate a QJT approach to analyze the sensed information for a better sensitivity: record the output of each autonomous QEC, count the number of single-photon jumps ($j\in \left\{0,1,...,M\right\}$), and process the data according to *j* [see Methods]. By doing so, ${{{{{{{\mathcal{Q}}}}}}}}$ is further improved in all cases and even the optimal *t*_int_ is extended, as shown in Fig. 3b–d. These experimental results demonstrate the protection and recovery of ${{{{{{{\mathcal{Q}}}}}}}}$ from decoherence by QEC and QJT, and indicate the benefits of approximate QEC in sensing.

### A radiometer enhanced by approximate QEC

**Fig. 4:**
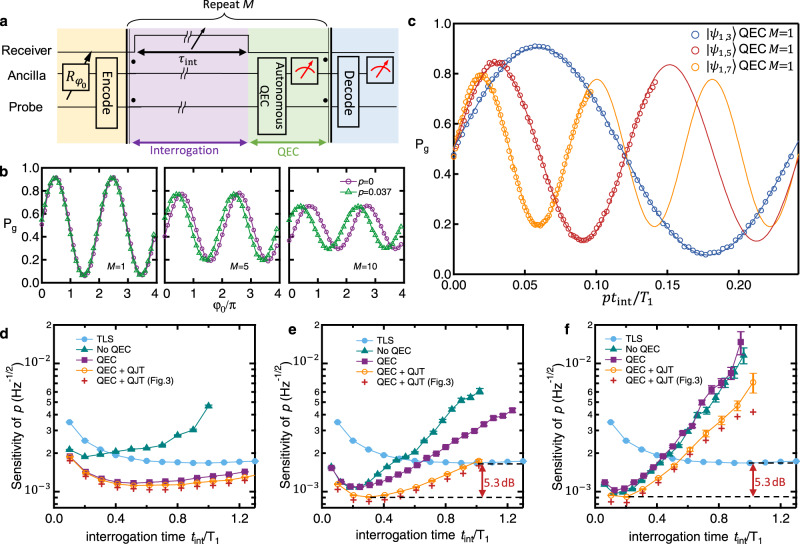
The quantum radiometry. **a** Experimental sequence for the quantum-enhanced radiometry that senses the excitation population *p* in the receiver cavity (Fig. 1c) via QEC. **b** The measured *P*_*g*_ as a function of the initial phase *φ*_0_. The purple dots and green triangles correspond to experiments with *p* = 0 and 0.037, respectively. The experiment is performed with the probe state $|{\psi }_{1,3}\rangle =(|1\rangle +|3\rangle )/\sqrt{2}$ and *τ*_int_ = 0.1*T*_1_ for the QEC repetition number *M* = 1, 5, 10 (from left to right). **c** The measured *P*_*g*_ as a function of *pt*_int_/*T*_1_ for the probe states $|{\psi }_{1,3}\rangle$, $|{\psi }_{1,5}\rangle$, and $|{\psi }_{1,7}\rangle$ with a single round of QEC (*M* = 1 and *t*_int_ = *Mτ*_int_ = *τ*_int_). The fitted oscillation periods are proportional to *n* − *m*. **d**–**f** Sensitivity of measuring *p* (*σ*_*p*_) of the radiometry for the probe states $|{\psi }_{1,3}\rangle$, $|{\psi }_{1,5}\rangle$, and $|{\psi }_{1,7}\rangle$, respectively. A sensitivity enhancement of 5.3 dB over TLS (the encoding with the two lowest Fock states) is obtained. QEC+QJT (Fig. 3): the deduced sensitivity from the results with the QEC+QJT strategy in Fig. 3. The error bars are obtained through error propagation of the fit parameter uncertainties.

Finally, the scheme is applied in a practical sensing scenario as a quantum radiometer. Based on the device shown in Fig. 1c and using the readout cavity as a receiver to the microwave field under detection, the excitation population of the readout cavity *p* = *ω*/*χ* could be derived by the quantum sensor with a calibrated cross-Kerr coefficient *χ* between the probe and the receiver. Following the sequence shown in Fig. 4a, the resulting oscillations shift due to the acquired phase *φ* from the receiver population *p* = 0.037 induced by a continuous weak coherent signal [Fig 4b]. It is observed that *φ* increases linearly with the acquisition time *Mτ*_int_ as expected, but the contrast of the signal fades due to decoherence and errors that cannot be completely corrected. With an appropriate *φ*_0_, the sensitivity of the output *P*_*g*_ could be maximized by optimizing the slope ∂*P*_*g*_/∂*p* for *p* ≈ 0. The results in Fig. 4c for different $|{\psi }_{m,n}\rangle$ show the maximum slopes around *p* ≈ 0 proportional to *n* − *m*. From these results, the achieved experimental sensitivity for measuring *p* is derived as

4 $${\sigma }_{p}={{\Delta }}{P}_{g}\sqrt{{t}_{{{{{{{{\rm{tot}}}}}}}}}}/| \partial {P}_{g}/\partial p| ,$$

with ${{\Delta }}{P}_{g}=\sqrt{{P}_{g}(1-{P}_{g})}\approx \frac{1}{2}$ being the standard deviation of *P*_*g*_ that follows the binomial statistics. When QJT is applied, *σ*_*p*_ could be derived with *P*_*g*_ and its slope conditional on *j* [see Methods]. Figure 4d–f summarizes the achieved sensitivities of the quantum radiometry for different strategies, as well as the potentially achievable ${\sigma }_{p}=1/\chi \sqrt{{{{{{{{\mathcal{Q}}}}}}}}}$ based on the virtual phase measurement results in Fig. 3. It is confirmed that the best strategy is to combine QEC and QJT and shows great advantage over others, with the sensitivity ${\sigma }_{{\psi }_{1,3}}=11.2$, ${\sigma }_{{\psi }_{1,5}}=9.1$, and ${\sigma }_{{\psi }_{1,7}}=9.1$ (in the unit of 10^−4^ Hz^−1/2^) achieved for $|{\psi }_{1,3}\rangle$, $|{\psi }_{1,5}\rangle$, and $|{\psi }_{1,7}\rangle$, respectively. The general trends of the achieved *σ*_*p*_ agree well with the results deduced from Fig. 3, but with a slight sensitivity loss due to the *j*-independent decoding in the current experiment instead of the most optimal adaptive decoding. Compared with the TLS case (*σ*_TLS_ = 16.7 × 10^−4^ Hz^−1/2^), we realize a sensitivity enhancement of $20{\log }_{10}{\sigma }_{{{{{{{{\rm{TLS}}}}}}}}}/{\sigma }_{{\psi }_{1,7}}=5.3\,{{{{{{{\rm{dB}}}}}}}},$ approaching the optimal enhancement of 6.2 dB implied by the results from Fig. 3.

The current sensitivity is mainly limited by the ancilla relaxation and dephasing errors, which are the dominant error source for achieving high-fidelity approximate QEC. The ancilla errors can be modeled as a depolarization channel for the QEC and their influence on the sensitivity can be numerically analyzed, which shows that the sensitivity can have another 3 dB improvement when the ancilla-induced depolarization probability decreases to 1/3 of the current value [see Supplementary Note 1]. It is worth noting that even provided with perfect quantum operations and an ideal ancilla, the optimal normalized QFI ${{{{{{{\mathcal{Q}}}}}}}}\propto n$, when *n* ≫ *m* (the Heisenberg limit ∝ *n*^2^ could not be achieved as pointed out earlier), and the signal-induced dephasing of the probe would further reduce ${{{{{{{\mathcal{Q}}}}}}}}$ for large *n* ~ 100 [see Supplementary Note 1].

## Discussion

In summary, utilizing the large Hilbert space of the bosonic mode, we realize a radiometry with a sensitivity of 9.1 × 10^−4^ Hz^−1/2^ in a superconducting circuit that shows a quantum enhancement of 5.3 dB and opens the door to practical quantum sensing. Our radiometry has a comparable sensitivity to the recently demonstrated thermometry for detecting propagating microwaves^35^ and the radiometry for sensing microwave radiation based on the photon-induced dephasing process of a superconducting qubit^36^. The gain of QFI by approximate QEC and QJT reveals the significant difference between the quantum sensing and other quantum information processing applications: the goal is to acquire the sensing information as much as possible instead of pursuing the perfect protection of an unknown quantum state. The extensions of the scheme to tens of photons by developing sophisticated quantum control methods and optimal approximate QEC codes, as well as to multiple bosonic modes, are appealing and worth further investigation. The bosonic radiometry demonstrated here will also excite immediate interest for other quantum sensing applications, such as the force sensing^37^, because the scheme is applicable to all physical quantities that could induce a change of *ω*. The bosonic probe having the advantages of hardware efficiency and avoiding the non-local interactions is extensible to other bosonic degrees of freedom including phonons coupled with trapped ions^10,38^ and superconducting qubits^39^, and also extensible to the collective excitations in spin and atom ensembles to promote the atomic and optical quantum metrology technologies^2–6^.

## Methods

### Experimental implementation

As described in the main text, our experiments are implemented with a superconducting quantum circuit and the device consists of a superconducting transmon qubit as an ancilla dispersively coupled to two superconducting rectangular microwave cavities, the probe, and the receiver. The input and output couplings of the receiver cavity are designed to be asymmetric, *κ*_r,out_ ≫ *κ*_r,in_, offering a decay rate of *κ*_r_ = 1/44 ns, which is three orders of magnitude higher than that of the probe cavity. As a consequence, a static coherent state with a mean excitation number *p* in the receiver cavity can be created within a negligible period of time. Such a design of coupling is fit for the high-fidelity readout of the ancilla. However, for practical applications, the receiver mode could be over-coupled to the external fields that are to be detected and an extra readout resonator could be employed to perform the readout. Crucially, the cross-Kerr interaction induced by the nonlinearity of the transmon allows the detection of the excitation in the receiver by utilizing the bosonic quantum states in the probe cavity. The calibrated cross-Kerr coefficient is *χ*/2*π* = 15.3 kHz.

### Quantum Fisher information (QFI)

To characterize the sensitivity that can be achieved in the experiment, the measured data are fitted with sinusoidal curves. For the simplest case with binary outputs $\{|g\rangle ,|e\rangle \}$, the results obey ${P}_{g}(\omega )=A+B\cos [\omega (n-m){t}_{{{{{{{{\rm{int}}}}}}}}}+{\varphi }_{0}]$ and *P*_*e*_ = 1 − *P*_*g*_. The final readout outputs follow the binomial distribution, giving a variance of ${({{\Delta }}{P}_{g})}^{2}={P}_{g}(\omega )-{P}_{g}^{2}(\omega )$. Therefore, the attainable resolution (uncertainty Δ*ω*) for measuring *φ* by one round of the sensing experiment, including the initialization, encoding, interrogation, decoding, and readout, reads

5 $${{\Delta }}\omega \ge {\min }_{{\varphi }_{0}}\frac{{{\Delta }}{P}_{g}}{\partial {P}_{g}\left(\omega \right)/\partial \omega }=\frac{\sqrt{A(1-A)}}{(n-m){t}_{{{{{{{{\rm{int}}}}}}}}}B}.$$

Alternatively, the precision could be studied in a more general frame of quantum metrology, where the QFI gain ${{{{{{{\mathcal{F}}}}}}}}$ could be deduced as

6 $$\hskip3.5pc{{{{{{{\mathcal{F}}}}}}}}={\max }_{{\varphi }_{0}}\left\{\frac{1}{{P}_{g}(\omega )}{\left[\frac{\partial {P}_{g}\left(\omega \right)}{\partial \omega }\right]}^{2}+\frac{1}{{P}_{e}(\omega )}{\left[\frac{\partial {P}_{e}\left(\omega \right)}{\partial \omega }\right]}^{2}\right\}$$

7 $$ =\frac{{(n-m)}^{2}{t}_{{{{{{{{\rm{int}}}}}}}}}^{2}{B}^{2}}{A\left(1-A\right)}.$$

The corresponding achievable measurement uncertainty is

8 $${{\Delta }}\omega \ge 1/\sqrt{{{{{{{{\mathcal{F}}}}}}}}}.$$

Introducing the normalized QFI per unit time ${{{{{{{\mathcal{Q}}}}}}}}={{{{{{{\mathcal{F}}}}}}}}/T$, with *T* being the experimental time, we then could derive the practical sensitivity of the experiment $1/\sqrt{{{{{{{{\mathcal{Q}}}}}}}}}$ that is associated with the measurement bandwidth and corresponds to the noise floor of the sensing. For example, if the duration for a single-shot sensing experiment is *t*_tot_, the QFI gain rate could be obtained as

9 $${{{{{{{{\mathcal{Q}}}}}}}}}_{\omega }=\frac{{B}^{2}{\left(n-m\right)}^{2}{t}_{{{{{{{{\rm{int}}}}}}}}}^{2}}{A(1-A){t}_{{{{{{{{\rm{tot}}}}}}}}}},$$

corresponding to the achievable measurement sensitivity

10 $${\sigma }_{\omega }\ge \frac{1}{\sqrt{{{{{{{{{\mathcal{Q}}}}}}}}}_{\omega }}}.$$

### Parameter optimization

For the probe quantum states, the damping channel could be represented by the Kraus operators as ${{{{{{{\mathcal{E}}}}}}}}\left(\rho \right)=\mathop{\sum }\nolimits_{k = 0}^{\infty }{E}_{k}\rho {E}_{k}^{{{{\dagger}}} }$, where *ρ* is the density matrix of the probe and

11 $${E}_{k}=\frac{{(1-{e}^{-\frac{{t}_{{{{{{{{\rm{int}}}}}}}}}}{{T}_{1}}})}^{k/2}}{\sqrt{k!}}{e}^{-\frac{{t}_{{{{{{{{\rm{int}}}}}}}}}}{2{T}_{1}}{a}^{{{{\dagger}}} }a}{a}^{k}$$

is the operator for the *k*-photon-loss error during a sensing interrogation time of *t*_int_. In the experiments, we only consider the two dominant errors $\left\{{E}_{0},\,{E}_{1}\right\}$. Due to the Fock state damping ${e}^{-\frac{{t}_{{{{{{{{\rm{int}}}}}}}}}}{2{T}_{1}}{a}^{{{{\dagger}}} }a}$ and the photon-number-dependent photon jump rate, the amplitudes of the two-component Fock states experience unbalanced amplitude change. Therefore, we numerically optimize the coefficients *α*_*m*,*n*_ and *β*_*m*,*n*_ of the initial probe quantum states, *τ*_int_, and the repetition number *M* to maximize ${{{{{{{\mathcal{Q}}}}}}}}$ by considering the full damping channel and the imperfections of QEC and other operations. We further optimize the interrogation time *τ*_int_ experimentally in order to acquire the maximum ${{{{{{{\mathcal{Q}}}}}}}}$. For the purpose of achieving the maximum sensitivity of the radiometry, we also optimize the initial phase *φ*_0_ in the encoding step to maximize the slope ∂*P*_*g*_/∂*p*.

### Autonomous implementation of QEC

The probe quantum states studied in this work are the approximate QEC codes, and thus the recovery ${R}_{j}=\left|m\right\rangle \left\langle m-j\right|+\left|n\right\rangle \left\langle n-j\right|+{\widetilde{R}}_{j}$ for the *j*-photon-loss error are derived according to the transpose channel, which provides a universal approach for constructing the recovery operation with reasonable performance. Such recovery operations are implemented autonomously and repetitively during the sensing. Specifically, a unitary operation *U* is implemented on the joint state of the probe and the ancilla qubit to transfer the error entropy associated with the encoded probe state to the ancilla: once a single-photon-loss error (*E*_1_) occurs, the probe state is recovered and the ancilla is flipped to the excited state $\left|e\right\rangle$. However, when there is no error, both the probe and the ancilla remain unaltered during the QEC operation. Explicitly, we have:

12 $$U\left|{\psi }_{{{{{{{{\rm{E}}}}}}}}}\right\rangle \left|g\right\rangle =\left|{\psi }_{{{{{{{{\rm{C}}}}}}}}}^{\prime}\right\rangle \left|e\right\rangle ,$$

13 $$U\left|{\psi }_{{{{{{{{\rm{C}}}}}}}}}\right\rangle \left|g\right\rangle =\left|{\psi }_{{{{{{{{\rm{C}}}}}}}}}\right\rangle \left|g\right\rangle ,$$

where $|{\psi }_{{{{{{{{\rm{E}}}}}}}}}\rangle$ is the erroneous state in the error space, $|{\psi }_{{{{{{{{\rm{C}}}}}}}}}\rangle$ is the uncorrupted state in the code space, and $|{\psi }_{{{{{{{{\rm{C}}}}}}}}}^{\prime}\rangle$ presents the recovered state with deformation (dashed arrows in Fig. 1a). Such a unitary operation is realized by sending numerically optimized microwave pulses simultaneously to both the probe cavity and the ancilla qubit, which are obtained through the so-called gradient ascent pulse engineering method based on the calibrated system parameters^40,41^.

After the autonomous implementation of QEC, the ancilla is then reset to the ground state to dump the error entropy for the next round of autonomous QEC. The reset of the ancilla could be performed in two ways. (1) The measurement-feedback method: the ancilla is first measured; the ancilla is left as it is if the measured outcome is the ground state, otherwise, it is flipped to the ground state by a feedback pulse. (2) The engineered-dissipation method: the ancilla is engineered to couple to a lossy channel, e.g., a cavity with a short lifetime, which allows a fast decay of the ancilla excitation regardless of the ancilla state, i.e., the ancilla state is not recorded. In our experiment, we use the measurement-feedback method to reset the ancilla, the ancilla state is recorded in each round of the QEC, and the outcome is used in the QJT protocol for extracting the sensitivity of our radiometry. The whole correction process is repeated for *M* times followed by a decoding for the final readout to end the sensing experiment.

Note that in contrast to the conventional QEC that consists of both error detection (a measurement of the ancilla) and measurement outcome-dependent error correction, the implementation of the autonomous QEC does not require error detection and fast real-time adaptive control. Therefore, such a protocol can avoid fast feedback electronics and also circumvent the delay in the electronics, and thus saves the hardware and suppresses potential decoherence due to the delay. For more information about the autonomous QEC, see Refs. ^33,34^.

### Quantum jump tracking (QJT)

We first note that here QJT refers to the specific approach to analyze the quantum sensing scheme by taking into account the number of jumps in each trajectory based on the implementation of autonomous QEC. The ancilla output of $\left|e\right\rangle$ after the QEC pulse indicates a single-photon-loss error occurs during the interrogation time. In the QJT experiments, the number of $\left|e\right\rangle$ at the output of the QEC pulse is counted and recorded, which allows us to improve the sensitivity. In general, the decoding operation should be adaptively selected according to the number of the quantum jumps (*j*). The optimal decoding scheme is to map the Fock states $\left|m\right\rangle$ and $\left|n\right\rangle$ to the ancilla state $|\pm \rangle =|g\rangle \pm {e}^{\pm i{\varphi }_{1}}\left|e\right\rangle$ for all cases, with *φ*_1_ ≈ 0 being the readout phase, therefore we only selectively process the data after the experiments. We divide the final measurement outputs into groups by *j*. The joint probability of output $\left|g\right\rangle$, *P*_*g*,*j*∣*M*_(*ω*), for *j* jumps, corresponding to the case with the single-photon-loss error occurring *j* times among *M* repetitions of sensing, can be fitted with ${P}_{g,j| M}(\omega )={A}_{g,j}+{B}_{g,j}\cos [\omega (n-m){t}_{{{{{{{{\rm{int}}}}}}}}}+{\varphi }_{g,j}]$. Similarly, for output $\left|e\right\rangle$, *P*_*e*,*j*∣*M*_(*ω*) can be fitted with ${P}_{e,j| M}(\omega )={A}_{e,j}+{B}_{e,j}\cos [\omega (n-m){t}_{{{{{{{{\rm{int}}}}}}}}}+{\varphi }_{e,j}]$. Here, *φ*_*g*(*e*),*j*_ includes the initial and the readout phases. The resulting normalized QFI ${{{{{{{\mathcal{Q}}}}}}}}$ with QJT can be calculated as

14 $${{{{{{{{\mathcal{Q}}}}}}}}}_{\omega } =	\frac{1}{{t}_{{{{{{{{\rm{tot}}}}}}}}}}{\max }_{{\varphi }_{0}}\left\{\mathop{\sum }\limits_{j=0}^{M}\mathop{\sum}\limits_{l\in \left\{g,e\right\}}\frac{1}{{P}_{l,j| M}(\omega )}{\left[\frac{\partial {P}_{l,j| M}(\omega )}{\partial \omega }\right]}^{2}\right\}\\ =	\frac{1}{{t}_{{{{{{{{\rm{tot}}}}}}}}}}\mathop{\sum }\limits_{j=0}^{M}\mathop{\sum}\limits_{l\in \left\{g,e\right\}}\frac{{B}_{l,j}^{2}{(n-m)}^{2}{t}_{{{{{{{{\rm{int}}}}}}}}}^{2}}{{A}_{l,j}}.$$

The experimental ${{{{{{{{\mathcal{Q}}}}}}}}}_{\omega }$ is calculated by the coefficients *A*_*j*_ and *B*_*j*_, which are obtained in the experiments of measuring the virtual phase by varying *φ*_0_. The results indicate a sensitivity of measuring *p* as

15 $${\sigma }_{p}=\frac{1}{\sqrt{{{{{{{{{\mathcal{Q}}}}}}}}}_{\omega }}}/\frac{\partial \omega }{\partial p}=\frac{1}{\sqrt{{{{{{{{{\mathcal{Q}}}}}}}}}_{\omega }}}\frac{1}{\chi }.$$

For the direct implementation of the radiometry, the sensitivity of measuring *p* is provided as

16 $${\sigma }_{p}=\frac{1}{\sqrt{{{{{{{{{\mathcal{Q}}}}}}}}}_{p}}},$$

with

17 $${{{{{{{{\mathcal{Q}}}}}}}}}_{p}=\frac{1}{{t}_{{{{{{{{\rm{tot}}}}}}}}}}\mathop{\sum }\limits_{j = 0}^{M}\mathop{\sum }\limits_{l\in \left\{g,e\right\}}\frac{1}{{P}_{l,j| M}}{\left[\frac{\partial {P}_{l,j| M}}{\partial p}\right]}^{2},$$

where the probabilities *P*_*l*,*j*∣*M*_ and their slopes $\frac{\partial {P}_{l,j| M}}{\partial p}$ are obtained directly from experiments with an optimized *φ*_0_.

## Supplementary information

Supplementary Information

Description of Additional Supplementary Information

Supplementary Data

## Data Availability

All data relevant to this study are available from the corresponding authors upon reasonable request. Source Data are provided with this paper.

## Source data

Source Data

## Code availability

The code used for simulations is available from the corresponding authors upon reasonable request.
