# Supplementary material for: Quantum-enhanced radiometry via approximate quantum error correction
Source: Nat Commun. 2022 Jun 9;13:3214. doi: 10.1038/s41467-022-30410-8 (PMC9184621; doi:10.1038/s41467-022-30410-8)
Supplement: Supplementary file 1 — Supplementary Information [file 41467_2022_30410_MOESM1_ESM.pdf]

Supplementary Information for "Quantum-enhanced radiometry via approximate quantum error correction"

W. Wang et al.

I. SUPPLEMENTARY NOTE 1 - THEORY

A. Basics about quantum metrology

In a quantum metrology experiment, a probe state is initialized as ρ, and then evolves in the environment associated with the parameter ω under detection, i.e. $\rho \rightarrow \rho_\omega = \mathscr{E}_\omega(\rho, t)$, with $\mathscr{E}_\omega$ denoting the quantum channel and t being the duration of the evolution. Quantum Cramer-Rao bound is one of the most fundamental limits in such a quantum metrology experiment and determines the best precision for the estimation of the parameter with an unbiased estimator [1]

$$\Delta\omega \geq 1/\sqrt{\mathscr{F}}, \tag{1}$$

where $\mathscr{F}$ is the quantum Fisher information (QFI). QFI is the maximum classical Fisher information that could be achieved with an optimal measurement. It can be calculated as [1]

$$\mathscr{F} = 8 \lim_{\delta\omega\rightarrow 0} \frac{1 - \mathrm{Tr}\sqrt{\sqrt{\rho_\omega}\rho_{\omega+\delta\omega}\sqrt{\rho_\omega}}}{\delta\omega^2}. \tag{2}$$

Supposing the output probability distribution of the optimal measurement is $\{P_i\}$, QFI can then be calculated as

$$\mathscr{F} = \sum_i \frac{1}{P_i}\left(\frac{\partial P_i}{\partial\omega}\right)^2. \tag{3}$$

The detailed calculation for our scheme is provided in the Methods in the main text.

B. Ideal model

The practical quantum-enhanced sensing in our experiment is implemented based on the cross-Kerr effect between a receiver cavity and a probe cavity, and the dominant error in the detection is the amplitude damping of the probe. Therefore, we can simplify the model as the estimation of ω under the amplitude damping noise. The evolution of the probe could be described by the master equation ($\hbar = 1$)

$$\frac{d}{dt}\rho = -i\omega\left[a^\dagger a, \rho\right] + \frac{1}{2T_1}\mathscr{L}_a(\rho), \tag{4}$$

where a is the annihilation operator of the bosonic mode, T_1 is the energy relaxation time of the bosonic mode, and

$$\mathscr{L}_o(\rho) = 2o\rho o^\dagger - \rho o^\dagger o - o^\dagger o\rho \tag{5}$$

is the Lindblad super-operator for a jump operator o. The evolution of the quantum state can also be described by the Kraus representation as

$$\mathscr{E} \circ U_\omega(\rho) = \sum_{k=0}^{\infty} E_k e^{-i\omega t a^\dagger a}\rho e^{i\omega t a^\dagger a} E_k^\dagger, \tag{6}$$

where $U_\omega(\rho) = e^{-i\omega t a^\dagger a}\rho e^{i\omega t a^\dagger a}$ is the unitary evolution with the estimated parameter ω and

$$E_k = \frac{(1 - e^{-\frac{t_{\mathrm{int}}}{T_1}})^{k/2}}{\sqrt{k!}} e^{-\frac{t_{\mathrm{int}}}{2T_1}a^\dagger a} a^k \tag{7}$$

is the operator for the k-photon-loss error during a sensing interrogation time of t_{int}.

As predicted theoretically by Zhou et al. [2], the ultimate Heisenberg limit is achievable via quantum error correction (QEC) when the interaction Hamiltonian and the decoherence effect (Lindblad form) satisfy the so-called "Hamiltonian-not-in-Lindblad span" (HNLS) condition. However, the model that we study in this work violates the HNLS condition, i.e. the sensing Hamiltonian $\omega a^\dagger a$ is subject to the Markovian noise described by the linear span of $\{I, a, a^\dagger, a^\dagger a\}$ and the signal cannot be separated from the noise since they are not orthogonal to each other. In this case, the Heisenberg limit cannot be achieved with a finite-dimensional probe state even provided with perfect quantum operation and noiseless ancillary qubits. However, although the ideal Heisenberg limit is not achievable, the protection of the probe from noise by QEC can still be advantageous in practical metrology applications.

1. Scheme with approximate QEC

As discussed above, we could not find an exact QEC code that is able to protect the quantum state from noise while preserving the accumulated sensing signal. Therefore, we resort to the approximate QEC codes [3] that could not perfectly correct the single-photon-loss errors. We choose the two-component Fock state superposition in the form $|\psi_{m,n}\rangle = \alpha_{m,n}|m\rangle + \beta_{m,n}|n\rangle$ as a probe to sense the parameter ω by accumulating the relative phase between the two Fock states. We note that the jump of a single-photon loss would not destroy the relative phase. For approximate QEC codes, a universal and near-optimal way to construct the QEC channel is based on the transpose channel method [3]:

$$\mathscr{R}(\rho) = \sum_{l=0}^{N} \Pi E_l^{\dagger} \mathscr{E}^{-1/2}(\Pi)\, \rho\, \mathscr{E}^{-1/2}(\Pi)\, E_l \Pi, \tag{8}$$

where Π is the projection operator onto the code space, and N is the number of independent Kraus operators corresponding to the noisy evolutions that we want to reverse. Such a channel provides a universal means to construct a perfect recovery operation without exhaustive numerical optimization if the Knill-Laflamme condition [4] is satisfied, otherwise it provides a high-fidelity QEC recovery, i.e. it realizes $\mathscr{R} \circ \mathscr{E} \approx I$.

Considering the two-component Fock state superposition with the projection operator onto the code space

$$\Pi = |n\rangle\langle n| + |m\rangle\langle m| \tag{9}$$

and the two major jump errors of the amplitude damping channel [Supplementary Eq. (7)] of $\{E_0, E_1\}$ with

$$E_0 = e^{-\frac{t_{\mathrm{int}}}{2T_1} a^{\dagger} a} = I - \frac{t_{\mathrm{int}}}{2T_1} a^{\dagger} a + O\left[\left(\frac{t_{\mathrm{int}}}{T_1}\right)^2\right], \tag{10}$$

$$E_1 = \sqrt{1 - e^{-\frac{t_{\mathrm{int}}}{T_1}}}\, e^{-\frac{t_{\mathrm{int}}}{2T_1} a^{\dagger} a} a = a\sqrt{\frac{t_{\mathrm{int}}}{T_1}} + O\left[\left(\frac{t_{\mathrm{int}}}{T_1}\right)^2\right], \tag{11}$$

we have a simple recovery channel constructed by the transpose channel method as

$$\mathscr{R}_0 = |m\rangle\langle m| + |n\rangle\langle n| + \widetilde{\mathscr{R}}_0, \tag{12}$$

$$\mathscr{R}_1 = |m\rangle\langle m-1| + |n\rangle\langle n-1| + \widetilde{\mathscr{R}}_1, \tag{13}$$

where $\widetilde{\mathscr{R}_i}$ is a complementary operator to make $\mathscr{R}_i$ unitary. The recovery operators correspond to the following processes: if the probe state loses one photon during the interrogation time t_{int}, $\mathscr{R}_1$ will be performed and drive the state back to the code subspace; otherwise, $\mathscr{R}_0$ is performed that corresponds to no action to the code space. It is worth noting that the error syndrome can be detected by a parity measurement with an ancillary qubit, such as $e^{-i\frac{\pi}{2}a^{\dagger}a\sigma_x}$ that determines the even or odd parity of the mode with σ_x being the Pauli operator on the ancilla qubit [5].

2. Achievable QFI

To characterize the best achievable sensitivity by our scheme, we first assume the QEC can be performed perfectly with infinitely high frequency and infinitesimal execution time, and then the damping channel could be converted to an effective master equation [6]

$$\frac{d}{dt}\rho = -i\omega\left[a^{\dagger}a, \rho\right] + \frac{1}{2T_1}\mathscr{L}_{\sqrt{a^{\dagger}a}}(\rho). \tag{14}$$

By solving the equation with $\rho(t) = \sum_{j,k} \rho_{j,k}(t)|j\rangle\langle k|$, we obtain

$$\rho_{jk}(t) = \rho_{jk}(0)\exp\left\{\left[-i\omega(j-k) + \frac{1}{T_1}\sqrt{jk} - \frac{1}{2T_1}(j+k)\right]t\right\}. \tag{15}$$

Therefore, for the initial state $|\psi_{m,n}\rangle = \alpha_{m,n}|m\rangle + \beta_{m,n}|n\rangle$, the QFI can be calculated as

$$\mathscr{F} = 4|\alpha_{m,n}|^2|\beta_{m,n}|^2 t^2 (m-n)^2 e^{-(\sqrt{m}-\sqrt{n})^2 t/T_1}. \tag{16}$$

The optimal normalized QFI ($\mathscr{F}/t$)

$$\mathscr{Q}_\omega = \frac{T_1 (m-n)^2}{e\left(\sqrt{m}-\sqrt{n}\right)^2} \tag{17}$$

is achieved when the interrogation time $t_{\text{opt}} = \frac{T_1}{(\sqrt{m}-\sqrt{n})^2}$, and $\alpha_{m,n} = \beta_{m,n} = \sqrt{2}/2$.

To obtain the maximum QFI, the optimal measurement bases should be chosen as $|\phi_\pm\rangle = (|n\rangle \pm |m\rangle)/\sqrt{2}$. This can be proved as follows. The probabilities of measuring the final state Supplementary Eq. (15) along the basis states $|\phi_+\rangle$ and $|\phi_-\rangle$ are

$$\begin{aligned} P_+ =& \text{Tr}\left[|\phi_+\rangle\langle\phi_+|\rho_{mn}(t)\right] \\ =& A + B\cos\left[\omega(m-n)t - \varphi\right] \end{aligned} \tag{18}$$

and

$$P_- = 1 - P_+, \tag{19}$$

respectively. Here $A = \frac{1}{2}$, $B = |\alpha_{m,n}\beta_{m,n}|\exp\left[-\left(\sqrt{m}-\sqrt{n}\right)^2 \frac{t}{2T_1}\right]$, and $\varphi = \text{Arg}\left(\alpha_{m,n}\beta^*_{m,n}\right)$. Then the corresponding QFI is equal to the one we calculate in Supplementary Eq. (16) and can be derived as

$$\begin{aligned} \mathscr{F} =& \sum_{l=\pm} \frac{1}{P_l}\left(\frac{\partial P_l}{\partial \omega}\right)^2 \\ =& \frac{(m-n)^2 B^2 t^2}{A(1-A)} \qquad (20) \\ =& 4|\alpha_{m,n}|^2 |\beta_{m,n}|^2 (m-n)^2 t^2 e^{-(\sqrt{m}-\sqrt{n})^2 t/T_1}, \qquad (21) \end{aligned}$$

where the second equation is optimized with $\omega(m-n)t - \varphi = \frac{\pi}{2} + k\pi$ and k is an arbitrary integer.

3. *Quantum jump tracking scheme*

The QEC operations, i.e. Eqs. (12) and (13), could preserve the Fock state basis of the probe state while the amplitude is still influenced by the photon-jump errors, as a result of the approximate QEC codes. Different trajectories of the system with different numbers of photon jumps will give different amplitudes of the Fock states, and then the corresponding different pure states are mixed together giving a mixed output state. Thus, the interference fringes of the measurement of the relative phase degrade with time, even with perfect QEC operations. However, as we will see below that the QFI could be further enhanced if the number of jumps of different experimental trajectories is taken into account.

Supplementary Fig. 1 compares the probe cavity state evolutions with and without considering the photon jump trajectory. When an error projects the state from the code subspace into an orthogonal error subspace and the subsequent QEC recovers the state back to the code space, the code space will be deformed due to the photon-number-dependent amplitude decay, as illustrated in Fig. 1 of the main text. Therefore, by distinguishing the evolution trajectory of the quantum state and selectively performing operations on the state, we will get a pure state instead of a mixed state at the end of the experiment, which can enhance the final QFI. We call this method the quantum jump tracking (QJT) method. We will show how to apply this method in the above example and evaluate its effect on the enhancement.

It is known that QFI is convex [7]

$$\mathscr{F}\left(p_1\rho_{1,\omega} + p_2\rho_{2,\omega}\right) \le \mathscr{F}\left(p_1\rho_{1,\omega} \oplus p_2\rho_{2,\omega}\right) = p_1\mathscr{F}\left(\rho_{1,\omega}\right) + p_2\mathscr{F}\left(\rho_{2,\omega}\right). \tag{22}$$

Here notation "$\oplus$" is the direct sum and can be described in the matrix form as

$$p_1\rho_{1,\omega} \oplus p_2\rho_{2,\omega} = \begin{pmatrix} p_1\rho_{1,\omega} & \\ & p_2\rho_{2,\omega} \end{pmatrix}, \tag{23}$$

which represents two distinguishable states $\rho_{1,\omega}$ and $\rho_{2,\omega}$ of the system with probabilities p_1 and p_2, respectively. For an initial state $|\psi_{m,n}\rangle = \alpha_{m,n}|m\rangle + \beta_{m,n}|n\rangle$ with $m \ll n$, the evolution with different photon losses can be tracked in each QEC cycle with

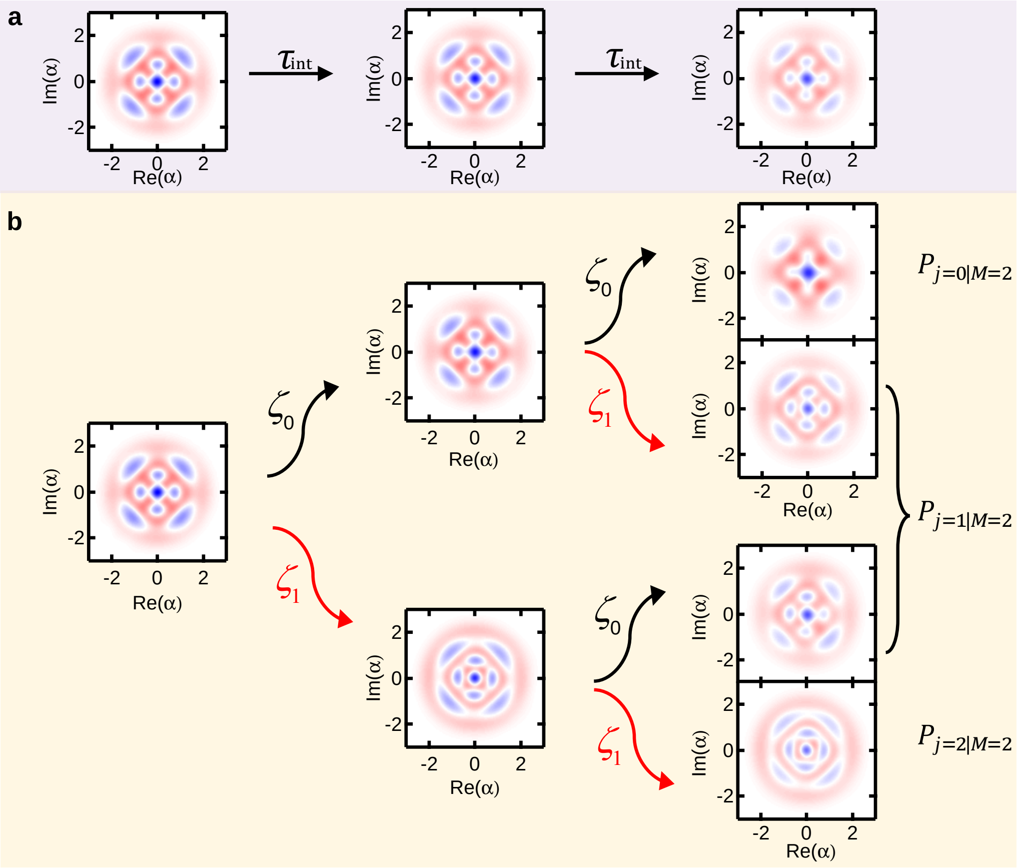

Supplementary Figure 1: **Schematic of the QJT scheme. a**, Simulated evolution of the Wigner function of the probe state $|\psi_{1,5}\rangle$. Due to decoherences, the state fidelity reduces as the interference fringes degrade. **b**, Possible trajectories of the simulated evolution of the Wigner function of the same probe state $|\psi_{1,5}\rangle$, with $\zeta_{0,1}$ denoting the jump with 0 or 1 photon-loss event. After two steps, there are four possible outputs with different trajectories. The weighted combination of the output with the corresponding probabilities gives the same result as that in (**a**). The parameters in the simulation refer to Supplementary Table 3.

the QJT method, therefore the final state can be described by the direct sum of states with different photon loss trajectories. With this description, the evolution with the QEC recycled for M times and an interrogation time $\tau_{\rm int}$ for each round becomes:

$$\begin{aligned}\rho &\rightarrow (\mathscr{R}\circ\mathscr{E}\circ U_\omega)^M(\rho)\\ &= \oplus_{l_1,\ldots l_M=0,1}\mathscr{R}_{l_M}E_{l_M}\ldots\mathscr{R}_{l_1}E_{l_1}e^{-i\omega M\tau_{\rm int}a^\dagger a}\rho e^{i\omega M\tau_{\rm int}a^\dagger a}E_{l_1}^\dagger\mathscr{R}_{l_1}^\dagger\ldots E_{l_M}^\dagger\mathscr{R}_{l_M}^\dagger\oplus Rest\\ &= \oplus_{x=0}^{M}C_M^x\left(e^{\tau_{\rm int}/T_1}-1\right)^x\left(a^\dagger a\right)^{x/2}e^{-\tau_{\rm int}a^\dagger a/2T_1}e^{-i\omega\tau_{\rm int}a^\dagger a}\rho e^{i\omega\tau_{\rm int}a^\dagger a}e^{-\tau_{\rm int}a^\dagger a/2T_1}\left(a^\dagger a\right)^{x/2}\oplus Rest.\end{aligned}\tag{24}$$

During the whole evolution, we assume there is at most one photon loss in each QEC cycle. The number of the trajectories with x-photon losses in total is the binomial coefficient C_M^x. It is worth noting that trajectories with the same number of photon losses produce the same final state and we can describe them by sum instead of direct sum without changing the final QFI. In addition, the total dimension of the first term ($\oplus_{x=0}^M$) is $(M+1)^2 s_\rho^2$ if the dimension of the initial state ρ is s_ρ^2. The term *Rest* represents the rest part of the whole states. For this direct sum state, the QFI can be calculated easily:

$$\mathscr{F}=\sum_{x=0}^{M}C_M^x\frac{4|\alpha_{m,n}|^2|\beta_{m,n}|^2(m-n)^2(mn)^x(M\tau_{\rm int})^2\left(e^{\tau_{\rm int}/T_1}-1\right)^x}{|\alpha_{m,n}|^2m^xe^{nM\tau_{\rm int}/T_1}+|\beta_{m,n}|^2n^xe^{mM\tau_{\rm int}/T_1}}+\mathscr{F}(Rest).\tag{25}$$

It can also be proved that the optimal measurement bases should be chosen as $|\phi_\pm\rangle=(|n\rangle\pm|m\rangle)/\sqrt{2}$. This method is similar to that in Supplementary Sec. I B 2. The enhanced normalized QFI as a function of the total interrogation time $t_{\rm int}=M\tau_{\rm int}$ is shown in Supplementary Fig. 2**a** and the numerical results with an ideal model prove the advantage of the QJT method.

C. Practical model

When it comes to the real experimental system, high-order photon jumps and operation errors should be taken into consideration. However, the GRAPE pulse for the QEC is complicated and makes it time-consuming to find the optimal initial probe state from simulation. In addition, the GRAPE pulse will not work perfectly in the presence of noise and operation errors, and will lead to the non-target states that are almost evenly distributed in the Hilbert space. We therefore approximate such a complex decoherence process as a depolarization channel in order to find the nearly optimal initial probe state without too much numerical calculation, i.e.

$$\mathscr{E}_{\mathrm{GRAPE}}\left(\rho\right)=\frac{\varepsilon_{\mathrm{op}}}{n_{\mathrm{tranc}}}I+\left(1-\varepsilon_{\mathrm{op}}\right)U\left(\rho\right). \tag{26}$$

Here n_{tranc} is the dimension of the truncated Fock space, and U is the target unitary operation that we want in the encoding, QEC, and decoding processes. The operation error probability, i.e. the depolarization probability, reads

$$\varepsilon_{\mathrm{op}}=1-\left(F_{\mathrm{GRAPE}}-\frac{1}{n_{\mathrm{tranc}}^2}\right)/\frac{n_{\mathrm{tranc}}^2-1}{n_{\mathrm{tranc}}^2}, \tag{27}$$

which is calculated according to the fidelity F_{GRAPE} of the GRAPE process.

Although we make a significant simplification of the experimental decoherence, our error model seizes the main characteristics of the decoherence and could provide a reasonable estimation of the system performance. With this simplified model, we could estimate the nearly optimal working point and explain the experimental result quantitatively, while avoiding massive numerical simulations.

D. Influence of ancilla imperfection

To evaluate the influence of the ancilla imperfections including both the relaxation and the dephasing effects of the transmon qubit, we numerically study the QFI of the system with different experimental parameters base on the practical model. As shown in Supplementary Fig. 2**b**, when including the ancilla-imperfection-induced operation errors, the QEC scheme may lead to a reduction of the normalized QFI since it may mistake the error state and "correct" it into a wrong subspace. In contrast, the QJT scheme is still robust. We note that such a toy model only provides a rough estimation of the scheme performances, and the numerical results of the different schemes with a full model without simplification are provided in Supplementary Sec. III.

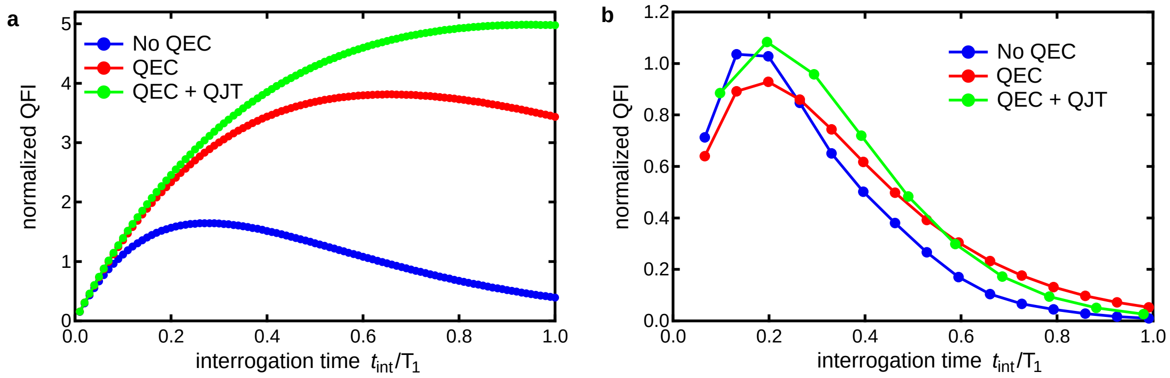

Supplementary Figure 2: **Normalized QFI as a function of the total interrogation time t_{int} for different schemes.** The initial probe state is $\left|\psi_{1,5}\right\rangle=\alpha_{1,5}\left|1\right\rangle+\beta_{1,5}\left|5\right\rangle$, where both $\alpha_{1,5}$ and $\beta_{1,5}$ are optimal corresponding to each scheme. **a**, The results with only the amplitude damping errors being considered during each interrogation time $\tau_{\mathrm{int}}=0.066T_1$. It shows the QEC and the QJT schemes can outperform the scheme without QEC. **b**, The results with amplitude damping errors, high-order photon jumps, initial state preparation error $\varepsilon_{\mathrm{ini}}=0.035$, operation errors $\varepsilon_{\mathrm{op}}=0.031$, and the measurement error $\varepsilon_{\mathrm{meas}}=0.038$ being considered. The decoherence parameters of the system in the simulation are consistent with the measured ones given in Supplementary Table 1. When these errors are included, the normalized QFI in the QEC scheme may be worse than the scheme without QEC, but the QJT scheme could still perform better.

To further reveal the potential limitation of the radiometry performance due to the ancilla imperfections, we simulate the practical model introduced above with different operation errors $x\times\varepsilon_{\mathrm{op}}$, by varying the scaling factor x, with $\varepsilon_{\mathrm{op}}=0.04$ according

to our experimental setup. Supplementary Fig. 3**a** plots the the enhancement factor $20\log_{10}(\mathscr{Q}/\mathscr{Q}_0)$, where $\mathscr{Q}_0$ is the normalized QFI with $x=1$ corresponding to the real experimental condition. In the simulation, the total interrogation time ($t_{\mathrm{int}} = M\tau_{\mathrm{int}} = 0.3T_1$) and the initial state ($|\psi_{1,5}\rangle = (|1\rangle+|5\rangle)/\sqrt{2}$) are fixed and the repetition round M is numerically optimized to get the maximum achievable $\mathscr{Q}$. According to these results, as the operation error decreases, more frequent QEC operations are allowed and higher normalize QFI can be achieved. These results illustrate the potential improvement of our scheme by reducing the operation errors in practical experiments.

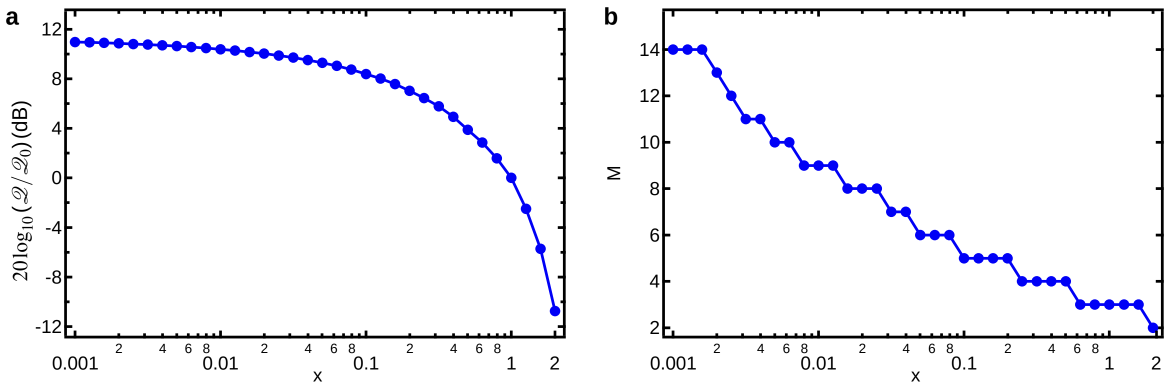

Supplementary Figure 3: **Prospect of the scheme performance with reduced operation errors.** **a**, Enhancement of the normalized QFI ($\mathscr{Q}$) compared with the real experimental one ($\mathscr{Q}_0$) with different operation error probabilities $x \times \varepsilon_{\mathrm{op}}$. **b**, The corresponding optimal repetition number M for data in (**a**). These two figures show the potential of our experimental system through the improvement of the operation errors, i.e. more frequent QEC operations and better normalized QFI can be obtained.

E. Signal-induced dephasing

The radiometry depicted in the main text includes the probe mode, the receiver mode, and the environment radiation that is to be detected. Although the excitations in the receiver mode would induce a frequency shift of the probe cavity, the fluctuation of the excitation numbers in the receiver would also induce a fluctuation of the probe frequency, and eventually gives rise to a dephasing effect of the probe cavity. In our current experimental setup, we investigate the accumulated phase of the probe cavity while neglecting the potential dephasing effect. In the following, we provide a detailed derivation to show that the dephasing effect is negligible for current experimental parameters.

The system could be described by the Hamiltonian

$$H = H_0 + H_1, \tag{28}$$

with the system's steady state interaction

$$H_0 = \chi p a^\dagger a + \left(\Omega_b + \frac{\chi}{2}\right) b^\dagger b + \sum_\Omega \Omega c_\Omega^\dagger c_\Omega + \sum_\Omega g_\Omega \left(c_\Omega b^\dagger + c_\Omega^\dagger b\right) \tag{29}$$

and fluctuations

$$H_1 = \chi\left(a^\dagger a - \frac{1}{2}\right)\left(b^\dagger b - p\right). \tag{30}$$

Here, the bosonic operators a, b and c_Ω denote the probe, the receiver, and the environment continuum modes, respectively. χ is the cross-Kerr coefficient between the probe and the receiver modes. p is the average excitation number induced by the environment radiation in the receiver (mode b). g_Ω denotes the coupling strength between the receiver mode and the continuum modes in the cable. Due to the quantum nature of radiations, the photon number fluctuation of the field $\left(b^\dagger b - p\right)$ would induce a decoherence effect on the probe. Here, we study this decoherence effect with our system parameters in the weak dispersion limit, i.e. $\chi \ll \kappa_b$ with κ_b being the amplitude decay rate of the receiver.

In the rotating frame of H_0, we have $\widetilde{b} = e^{iH_0t} b e^{-iH_0t}$ and $\widetilde{c}_\Omega = e^{iH_0t} c_\Omega e^{-iH_0t}$, and thus

$$\frac{\partial}{\partial t}\widetilde{b} = -i\sum_{\Omega} g_\Omega \widetilde{c}_\Omega - i(\Omega_b + \chi/2)\widetilde{b}, \tag{31}$$

$$\frac{\partial}{\partial t}\widetilde{c}_\Omega = -i\Omega\widetilde{c}_\Omega - ig_\Omega\widetilde{b}. \tag{32}$$

Under the white-noise approximation $g_\Omega = g_0$ and the Weisskopf-Wigner approximation, the receiver and the continuum coupling induces an external coupling loss rate $\kappa_b = \pi f_0 g_0^2$, with f_Ω being the density of states. Neglecting the intrinsic loss of the receiver cavity, we obtain the formal solution of the receiver as

$$\widetilde{b}(t) \simeq i\sum_{\Omega} \frac{g_\Omega \widetilde{c}_\Omega(0) e^{-i(\Omega-\Omega_b)t}}{i(\Omega - \Omega_b - \chi/2) - \kappa_b} \tag{33}$$

and the excitation number

$$\widetilde{b}^\dagger(t)\widetilde{b}(t) = \sum_{\Omega,\Omega'} \frac{g_\Omega g_{\Omega'} \widetilde{c}_\Omega^\dagger(0)}{-i(\Omega - \Omega_b - \chi/2) - \kappa_b} \frac{\widetilde{c}_{\Omega'}(0) e^{-i(\Omega'-\Omega)t}}{i(\Omega' - \Omega_b - \chi/2) - \kappa_b}. \tag{34}$$

For the probe state ρ_a, under the Born-Markov approximation, the evolution follows

$$\frac{\partial \rho_a}{\partial t} = -i\chi \mathrm{Tr}_R\left[\left(a^\dagger a - \chi/2\right)\left(\widetilde{b}^\dagger(t)\widetilde{b}(t) - p\right), \rho_a(0) \otimes \rho_R(0)\right] \tag{35}$$

$$-\chi^2 \mathrm{Tr}_R \int_0^\infty \left[\left(a^\dagger a - \chi/2\right)\left(\widetilde{b}^\dagger(t)\widetilde{b}(t) - p\right), \left[a^\dagger a\left(\widetilde{b}^\dagger(t-s)\widetilde{b}(t-s) - p\right), \rho_a(t) \otimes \rho_R(0)\right]\right] ds, \tag{36}$$

where the subscript R denotes both the system b and the continuum. For simplicity, we consider a component $\rho_{a,mn} = \langle m|\rho_a|n\rangle$ in the Fock basis following the evolution

$$\frac{\partial \rho_{a,mn}}{\partial t} = -\chi^2 \left\langle \int_0^\infty ds \left(\widetilde{b}^\dagger \widetilde{b}(t) - p\right)\left(\widetilde{b}^\dagger(t-s)\widetilde{b}(t-s) - p\right)\right\rangle \left[m^2 \rho_{a,mn} - 2mn\rho_{a,mn} + n^2 \rho_{a,mn}\right], \tag{37}$$

which corresponds to the master equation with a dephasing effect as

$$\frac{\partial \rho_a}{\partial t} = \gamma_{\mathrm{dph}} \mathscr{L}_{a^\dagger a}(\rho_a). \tag{38}$$

The corresponding probe cavity dephasing rate is

$$\gamma_{\mathrm{dph}} = \chi^2 \left\langle \int_0^\infty ds \left(\widetilde{b}^\dagger \widetilde{b}(t) - p\right)\left(\widetilde{b}^\dagger(t-s)\widetilde{b}(t-s) - p\right)\right\rangle. \tag{39}$$

Considering the case with the environment continuum modes centered at Ω_0 and an average excitation $p = \overline{n}_{\mathrm{coh}}$ and neglecting the thermal environment, we have

$$\gamma_{\mathrm{dph}} = \overline{n}_{\mathrm{coh}} \frac{\chi^2 \kappa_b}{\left[(\Omega_0 - \Omega_b - \chi/2)^2 + \kappa_b^2\right]}. \tag{40}$$

For the case with a thermal input to the receiver, which corresponds to a thermal equilibrium excitation number $p = \overline{n}_{\mathrm{th}}$, we have

$$\gamma_{\mathrm{dph}} = \left(\overline{n}_{\mathrm{th}}^2 + \overline{n}_{\mathrm{th}}\right)\frac{\chi^2}{\kappa_b}. \tag{41}$$

Comparing with the coherent phase accumulation rate χp, the dephasing rates γ_{dph} for both coherent and thermal signals in the receiver are negligible with our current experimental setup because $\chi/\kappa_b \ll 1$ and $\overline{n}_{\mathrm{th}} \ll 1$.

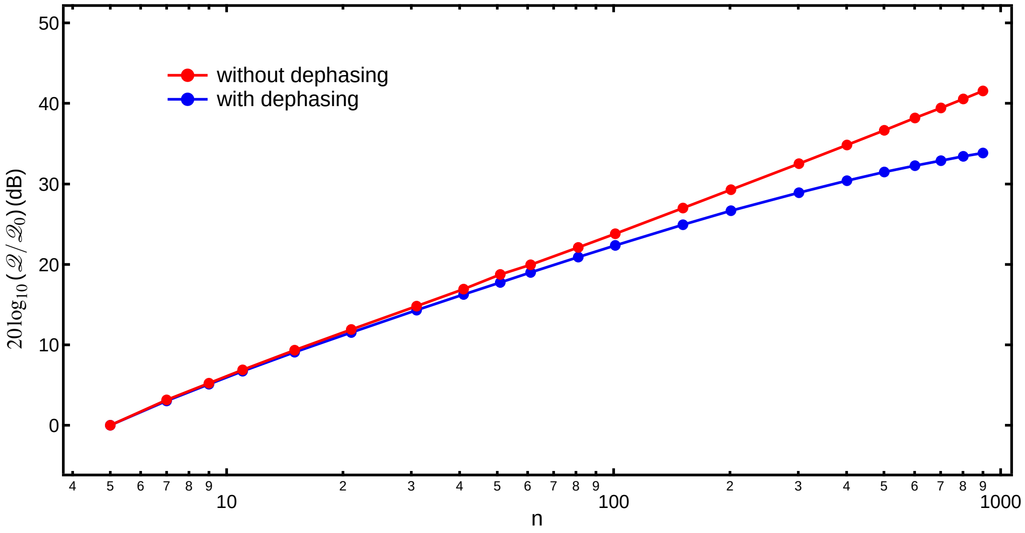

Supplementary Figure 4: **Influence of signal-induced dephasing with increasing n (with $m=1$).** The blue and the red points represent the QFI enhancement in a practical model with and without considering the signal-induced dephasing, respectively. $\mathscr{Q}_0$ is the reference value of the normalized QFI for $n=5$, with considering the signal-induced dephasing. The dephasing rate is calculated according to Supplementary Eq. (40), with $\bar{n}_{\mathrm{coh}}=0.01$ and other parameters from Supplementary Table 1.

F. Influence of signal-induced dephasing

As mentioned in the section above, signal could induce dephasing effect in the probe mode. Although the dephasing rate is negligible when compared with other decoherence rates, this effect could impose an ultimate limitation of the precision for our radiometry even in the ideal case. To show the influence of the signal-induced dephasing, we analytically calculate the approximate QEC scheme with continuous and perfect recovery operations. The evolution with the dephasing noise and recovery can be written as

$$\frac{d}{dt}\rho=-i\omega\left[a^{\dagger}a,\rho\right]+\frac{1}{2T_1}\mathscr{L}_{\sqrt{a^{\dagger}a}}(\rho)+\gamma_{\mathrm{dph}}\mathscr{L}_{a^{\dagger}a}(\rho), \tag{42}$$

where γ_{dph} could be evaluated by Supplementary Eq. (40) or Supplementary Eq. (41). By solving the equation with $\rho(t)=\sum_{j,k}\rho_{j,k}(t)\left|j\right\rangle\left\langle k\right|$, we obtain

$$\rho_{jk}(t)=\rho_{jk}(0)\exp\left\{\left[-i\omega(j-k)-\frac{1}{2T_1}\left(\sqrt{j}-\sqrt{k}\right)^2-\gamma_{\mathrm{dph}}(j-k)^2\right]t\right\}. \tag{43}$$

With the same initial state $\left|\psi_{m,n}\right\rangle=\frac{|m\rangle+|n\rangle}{\sqrt{2}}$ and the interrogation time $t_{\mathrm{opt}}=\frac{T_1}{(\sqrt{m}-\sqrt{n})^2}$, the final normalized QFI

$$\mathscr{Q}_{\omega}=\frac{T_1(m-n)^2}{e\left(\sqrt{m}-\sqrt{n}\right)^2}e^{-2\gamma_{\mathrm{dph}}(m-n)^2t_{\mathrm{opt}}}. \tag{44}$$

For $m=1$ and $n\gg1$, $\mathscr{Q}_{\omega}=\frac{T_1}{e}ne^{-2\gamma_{\mathrm{dph}}T_1n}$. Compared with the situation without the dephasing [Supplementary Eq. (17)], the signal-induced dephasing introduces an additional factor $e^{-2\gamma_{\mathrm{dph}}T_1n}$ to the normalized QFI. Therefore, the enhancement of the sensing by the non-trivial quantum state and the error correction will be eventually limited for large n.

The potential influence of the signal-induced dephasing in our practical system is further investigated by numerically studying the normalized QFI. In the practical model, the photon loss error of the probe mode, the imperfect operations due to the imperfect ancilla, and the signal-induced dephasing are considered. The results are summarized in Supplementary Fig. 4, with the input probe state being initialized as $\frac{|1\rangle+|n\rangle}{\sqrt{2}}$. Comparing the results for AQEC with and without the signal-induced dephasing, the QFI degrades due to the dephasing when n increases as expected. The signal-induced dephasing effect starts to be significant when $n\sim100$.

II. SUPPLEMENTARY NOTE 2 - EXPERIMENTS

ancilla qubit	$\omega_q/2\pi$	5.692 GHz
	T_1^q	30 μs
	T_ϕ^q	120 μs
	$K_q/2\pi$	232 MHz
probe cavity	$\omega_s/2\pi$	7.634 GHz
	T_1^s	143 μs
	T_ϕ^s	2.1 ms
	$K_s/2\pi$	4.23 kHz
	$K_s^{'}/2\pi$	0.45 kHz
receiver cavity	$\omega_r/2\pi$	8.610 GHz
	T_1^r	44 ns
	$K_r/2\pi$	(14.4 kHz)
interaction terms	$\chi_{qs}/2\pi$	1.90 MHz
	$\chi_{qr}/2\pi$	3.65 MHz
	$\chi_{sr}/2\pi$	15.3 kHz

Supplementary Table 1: Parameters of the experimental device. $\omega_{q,r,s}$, $T_1^{q,r,s}$ and $T_\phi^{q,s}$ are the transition frequency, energy relaxation time, and pure dephasing time, respectively. The subscripts and superscripts q, r, and s denote the transmon qubit, the receiver cavity, and the probe cavity, respectively. For simplicity, we have referred to T_1^s as T_1 in the main text and for the rest of Supplementary Information. χ_{qs}, χ_{qr}, χ_{sr} are the cross-Kerr coefficients. K_q, K_r, K_s, $K_s^{'}$ are the higher-order self-Kerr coefficients. All parameters are experimentally measured, except for K_r which is predicted through $\chi_{qr} = -2\sqrt{K_q K_r}$ according to the black-box quantization theory [8].

A. Experimental device and measured parameters

The experimental system consists of one ancillary transmon qubit and two three-dimensional cavities. The cavity with a long lifetime serves as the probe mode, while the other short-lived one is employed for fast high-fidelity readout of the qubit and particularly works as the receiver cavity for receiving the microwave signal from the outside in the sensing protocol. The Hamiltonian of the whole system can be written as

$$H/\hbar = \omega_q |e\rangle\langle e| + \omega_r a_r^\dagger a_r + \omega_s a_s^\dagger a_s - \chi_{qs} |e\rangle\langle e| a_s^\dagger a_s - \chi_{qr} |e\rangle\langle e| a_r^\dagger a_r \\ - \chi_{sr} a_s^\dagger a_s a_r^\dagger a_r - \frac{K_q}{2} a_q^{\dagger 2} a_q^2 - \frac{K_r}{2} a_r^{\dagger 2} a_r^2 - \frac{K_s}{2} a_s^{\dagger 2} a_s^2 - \frac{K_s^{'}}{6} a_s^{\dagger 3} a_s^3, \tag{45}$$

where $a_{q,s,r}$ are the annihilation operators of the ancilla qubit, the probe mode, and the receiver cavity, respectively; $\omega_{q,s,r}$ are the corresponding frequencies; χ_{qs}, χ_{qr}, χ_{sr} are the cross-Kerr coefficients; K_q, K_r, K_s, $K_s^{'}$ are the higher-order self-Kerr coefficients.

As demonstrated in the previous work [9], since the QEC operation is fast compared to the evolution time and the higher-order terms can be neglected, the above Hamiltonian can be approximated as

$$H_{qs}/\hbar = \omega_q|e\rangle\langle e| + \omega_s a_s^\dagger a_s + \omega_r a_r^\dagger a_r - \chi_{qs}|e\rangle\langle e| a_s^\dagger a_s - \chi_{qr}|e\rangle\langle e| a_r^\dagger a_r - \chi_{sr} a_s^\dagger a_s a_r^\dagger a_r. \tag{46}$$

In this system, the ancilla qubit dispersively couples to the photonic states in both the probe and the receiver cavities. With the assistance of the ancilla qubit, arbitrary unitary operation of the probe state can be implemented with selective photon-number-dependent arbitrary phase gate (SNAP) [10] or optimal quantum control by gradient ascent pulse engineering (GRAPE) [11, 12]. The interaction between the probe and the receiver modes allows the sensing protocol demonstrated in our work. In addition, the separation of probe and receiver provides the advantages of both longer coherence time and relatively larger bandwidth. The parameters of the experimental device are summarized in Supplementary Table 1. The detailed experimental setup can also be found in Ref. [13].

B. Characterization and calibration

In the main text, we experimentally demonstrate the QEC-enhanced quantum sensing of the excitation number (p) in an electromagnetic mode of the receiver cavity. This sensing relies on the cross-Kerr interaction χ_{sr} between the probe and the

receiver, and the final accumulated sensing signal on the probe state is then mapped to the ancilla that is measured at the end of the experiment. The cross-Kerr interaction χ_{sr} is obtained by measuring the receiver spectrum after deterministically preparing the Fock state $|n\rangle$ in the probe cavity. To increase the resolution in the regime of $\chi_{sr} \ll \kappa_r$, we prepare the Fock states $|n=0\rangle$ and $|n=10\rangle$ in the probe cavity respectively and obtain χ_{sr} from the frequency shift of the receiver. The measured $\chi_{sr} = 15.3$ kHz and this value agrees well with the estimated value based on the black-box quantization theory [8] with a deviation of 2%.

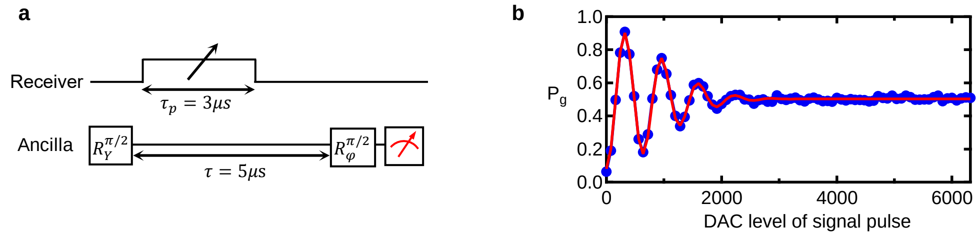

Supplementary Figure 5: **Calibration of the signal strength in the receiver cavity.** **a**, Pulse sequence for the photon-induced-dephasing measurement. Two $\pi/2$ qubit pulses are separated by $\tau = 5\ \mu$s, during which a square pulse with varied amplitudes for a duration of $\tau_p = 3\ \mu$s to excite the photonic field in the receiver cavity. τ_p is long enough for a steady state in the receiver cavity while the ancilla remains sufficiently coherent. By sweeping the phase difference φ of the two $\pi/2$ pulses, we can observe an oscillating interference signal of qubit population as a function of the signal strength. **b**, The ground state population P_g of the ancilla qubit as a function of the DAC level of the signal pulse. By fitting the envelope with a Gaussian function, The DAC level corresponding to a mean excitation number in the receiver mode $p = 1$ can be acquired.

In our demonstration, the signal from a typical radiative source is replaced by a coherent signal injected into the receiver cavity. The signal strength in the receiver cavity is calibrated by a photon-induced-dephasing process with the assistance of the ancilla qubit [14]. As shown in Supplementary Fig. 5**a**, the experimental sequence is a Ramsey-type measurement of the ancilla qubit inserted by a signal pulse with various amplitudes for a duration τ_p. We can observe Ramsey fringes with a decaying amplitude envelope $e^{-\tau\Gamma_2 - \tau_p\Gamma_p}$, where Γ_2 is the intrinsic dephasing rate, the photon-induced-dephasing rate $\Gamma_p = 2p\kappa_r \sin^2(\tan^{-1}(\chi_{qr}/\kappa_r))$, κ_r is the dissipation rate of the receiver, and χ_{qr} is the interaction between the qubit and the receiver. The measured probability of the qubit at the ground state P_g as a function of the signal pulse amplitude is shown in Supplementary Fig. 5**b**. Thus we can obtain a calibration of the signal strength p in the receiver cavity. We choose a long-enough duration of the signal to ensure the receiver is at the steady state while the ancilla qubit is still sufficiently coherent.

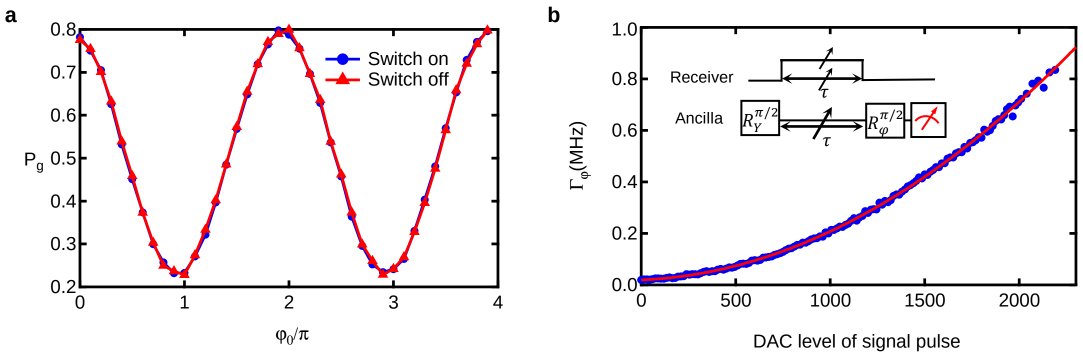

Supplementary Figure 6: **Calibration of photon-leakage in the receiver cavity.** **a**, The results of a Ramsey-like sensing experiment (same protocol as Fig. 2a in the main text). The blue dots and red triangles represent the qubit Ramsey-like oscillations (P_g for the probe state $|\psi_{1,3}\rangle = (|1\rangle + e^{i\varphi_0}|3\rangle)/\sqrt{2}$ against the initial phase φ_0 with an interrogation time $t_{int} = 0.2T_1$) with the digitized switch "on" but with nominally "zero" signal strength and "off" for completelly suppressing the leakage of the signal, respectively. **b**, The pure dephasing rate of the qubit Γ_φ as a function of the signal pulse amplitude in a photon-induced-dephasing experiment. The measurement protocol is shown in the inset. Blue dots show the experimental results and the red line represents a fit with a quadratic function, giving a leakage level of $p_{BG} = 8.8 \times 10^{-5}$.

Even though no photon is intentionally injected into the receiver externally, the external noise added to the receiver can be detected by the probe. The background noise in the receiver includes the thermal excitation by the environment and also the leakage from the signal source. We note that the input of the signal to the receiver cavity is controlled by a switch and the leakage can be completely suppressed by turning off the switch. To evaluate the influence of the signal leakage when the switch is on and the signal strength is nominally "zero" , we follow the sequence of Fig. 2**a** in the main text for a sensing test, we display two

oscillation curves in Supplementary Fig. 6**a**, both for $t_{\mathrm{int}} = 0.2T_1$ corresponding to the "on" (blue dots, with nominally "zero" signal strength) and "off" (red triangles) states of the switch, respectively. The comparison of these two curves shows the signal leakage is almost negligible.

To estimate the weak leakage more precisely, an alternative method is to measure the change of the pure dephasing rate of the ancilla qubit as the signal strength increases in a photon-induced-dephasing experiment (the protocol is similar to Supplementary Fig. 5**a** but also with a varying τ and is shown in the inset of Supplementary Fig. 6**b**). Supplementary Fig. 6**b** shows the pure dephasing rate Γ_φ as a function of the signal pulse amplitude. By fitting the curve with a quadratic function, we can acquire the signal leakage in the receiver cavity $p_{\mathrm{BG}} = 8.8 \times 10^{-5}$ when the derivative equals zero.

C. Parameters used in the experiment

n	$\alpha_{1,n}$	τ_{num}	τ_{exp}	$\tau_{\mathrm{exp}}^{\mathrm{w/o}}$
3	0.707	$0.100T_1$	$0.103T_1$	$0.100T_1$
5	0.699	$0.061T_1$	$0.062T_1$	$0.060T_1$
7	0.7	$0.063T_1$	$0.063T_1$	$0.060T_1$

Supplementary Table 2: The optimized coefficients $\alpha_{1,n}$ and the interrogation time τ_{int} for the photonic probe state of $|\psi_{1,\mathrm{n}}\rangle = \alpha_{1,n}|1\rangle + \beta_{1,n}|n\rangle$ used in the experiment with QEC. τ_{num} is the interrogation time obtained from the numerical optimization and τ_{exp} is the parameter applied in the experiment. $\tau_{\mathrm{exp}}^{\mathrm{w/o}}$ is the interrogation time in the experiment without QEC for the sake of comparison.

n	$\alpha_{1,n}$	$\tau_{\mathrm{num}}^{\mathrm{QJT}}$	$\tau_{\mathrm{exp}}^{\mathrm{QJT}}$
3	0.707	$0.100T_1$	$0.103T_1$
5	0.670	$0.098T_1$	$0.101T_1$
7	0.668	$0.097T_1$	$0.102T_1$

Supplementary Table 3: The optimized coefficients $\alpha_{1,n}$ and the interrogation time τ_{int} for the photonic probe state of $|\psi_{1,n}\rangle = \alpha_{1,n}|1\rangle + \beta_{1,n}|n\rangle$ used in the experiment with the QJT method.

The numerically optimized interrogation times τ_{int} are shown in Supplementary Table 2 and Supplementary Table 3 based on the simplified model in Sec. I C. In practice, the parameters used in the experiment could be further optimized according to the actual experimental performance. For comparison, the experiment without QEC is carried out with the interrogation time $t_{\mathrm{int}} = M\tau_{\mathrm{exp}}^{\mathrm{w/o}}$, where M is the number of the QEC repetitions.

D. Sensing with virtual phases

When implementing the experiment without QEC, the phase shift between the two components of the probe mode would accumulate for different evolution times owing to the Kerr effect. Such a Kerr effect induces a deterministic phase between different Fock states, and this resulted extra phase during the evolution is compensated in later experiments. The measured P_{g} for $|\psi_{1,3}\rangle$, $|\psi_{1,5}\rangle$, and $|\psi_{1,7}\rangle$ as a function of the initial phase φ_0 and the interrogation time t_{int} are displayed in Supplementary Figs. 7**a**, 7**c** and 7**d**, respectively. To show more clearly, the data in Fig. 2 of the main text have been adjusted by appropriate phase shifts, which are shown in Supplementary Fig. 7**b** and correspond to the Kerr effect for $n = 3$.

When the experiment with repeated QEC is carried out, the phase shifts due to the Kerr effect are corrected within the correction GRAPE pulse. Supplementary Figs. 8**a** and 8**b** show the measured P_{g} for $|\psi_{1,5}\rangle$ and $|\psi_{1,7}\rangle$ with repeated QEC, respectively. In this case, the phase shifts with different QEC repetition rounds are due to the non-perfect measurement-induced-phase compensation. As a result, before sensing the signal, it is necessary to figure out the optimal working point of the radiometry. For different total interrogation times t_{int}, the optimal working points φ_{opt} are chosen where the slope $\frac{dP_{\mathrm{g}}(\varphi_0)}{d\varphi_0}|_{\varphi_0=\varphi_{\mathrm{opt}}}$ is maximum.

In the QJT scheme, following the experimental sequence in Fig. 2**b** of main text, the result of each measurement is recorded. The ground state population of the ancilla $P_{\mathrm{g},j|M}$, corresponding to the trajectory with the single-photon-loss error occurring j times among M repetitions of QEC, can be fitted with $P_{\mathrm{g},j|M} = A_{g,j} + B_{g,j}\cos(\varphi_{g,j} + \varphi_0)$. Taking $|\psi_{1,3}\rangle = \alpha_{1,3}|1\rangle + \beta_{1,3}|3\rangle$ as an example, the results of first four repetitions have been shown in Supplementary Fig. 9. The normalized QFI for the QJT method can be calculated according to the experimentally measured Ramsey fringes, as shown in Fig. 3 of the main text. The processing procedure of the experimental data is provided in the Methods section of the main text.

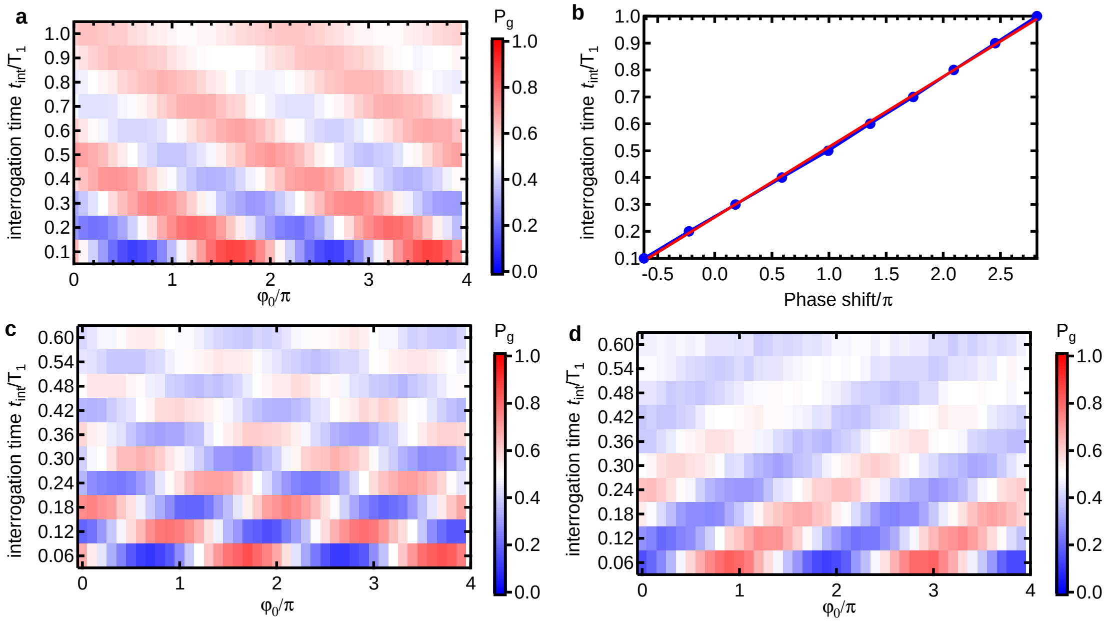

Supplementary Figure 7: **Measured P_g in the absence of QEC as a function of the initial phase φ_0 and the total interrogation time t_{int}.** **a**, P_g for the probe state $|\psi_{1,3}\rangle = \alpha_{1,3}|1\rangle + \beta_{1,3}|3\rangle$. **b**, The accumulated phase shift induced by the Kerr effect for $|\psi_{1,3}\rangle$. **c**, P_g for the probe state $|\psi_{1,5}\rangle = \alpha_{1,5}|1\rangle + \beta_{1,5}|5\rangle$. **d**, P_g for the probe state $|\psi_{1,7}\rangle = \alpha_{1,7}|1\rangle + \beta_{1,7}|7\rangle$. The parameters in these experiments are shown in Supplementary Table 2.

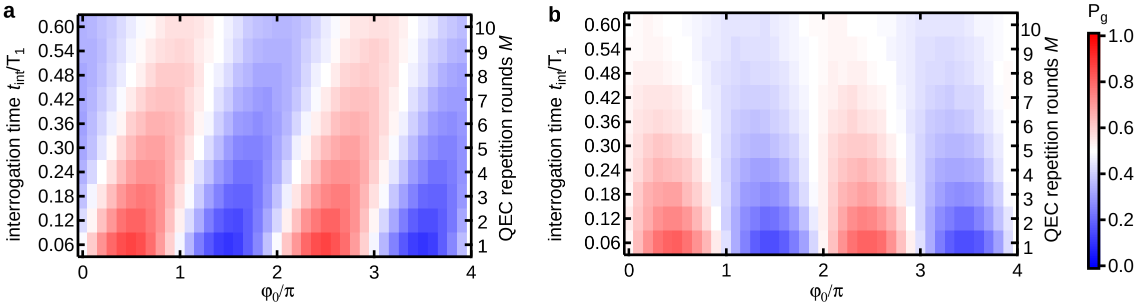

Supplementary Figure 8: **Measured P_g with QEC as a function of the initial phase φ_0 and the total interrogation time t_{int}.** **a** and **b**, The measured ground state population of the ancilla P_g for $|\psi_{1,5}\rangle = \alpha_{1,5}|1\rangle + \beta_{1,5}|5\rangle$ and $|\psi_{1,7}\rangle = \alpha_{1,7}|1\rangle + \beta_{1,7}|7\rangle$, respectively, with the parameters shown in Supplementary Table 2.

E. Radiometry protocol

In the following, we provide more details of our experimental sequences and results. Characterization of the sensitivity with QEC is carried out following the sequence shown in Supplementary Fig. 10**a**, while the sequence without QEC is displayed in Supplementary Fig. 10**b**. After encoding, the probe is set at the optimal working point with $\varphi_0 = \varphi_{opt}$, which is calibrated as described in Supplementary Sec. II D. Sensing the steady state of a coherent signal in the receiver is reflected on the phase accumulation between the two components of the bosonic probe, and such an accumulated phase is then decoded onto the qubit that is measured in the end.

Supplementary Fig. 11**a** shows the qubit ground population P_g as a function of the signal strength p in the receiver, where the probe state is $|\psi_{1,3}\rangle = (|1\rangle + |3\rangle)/\sqrt{2}$ with repeated QEC for $M = 4$ times and each interrogation time $\tau_{int} = 0.1T_1$. For comparison, the experiment in which the probe and the receiver interact for $t_{int} = 0.4T_1$ without QEC is also shown with a smaller oscillation amplitude. The interrogation time is optimized in the experiment and shown in Supplementary Table 2, followed by a short waiting time for the evacuation of the photons in the receiver to minimize their deleterious effect on the QEC process.

Supplementary Fig. 11**b** shows the sensing signal for $|\psi_{1,3}\rangle$ with QEC for different repetition rounds M. The oscillation frequency of $P_g(p)$ increases with more repetitions, while the contrast of the signal diminishes. For different probe states,

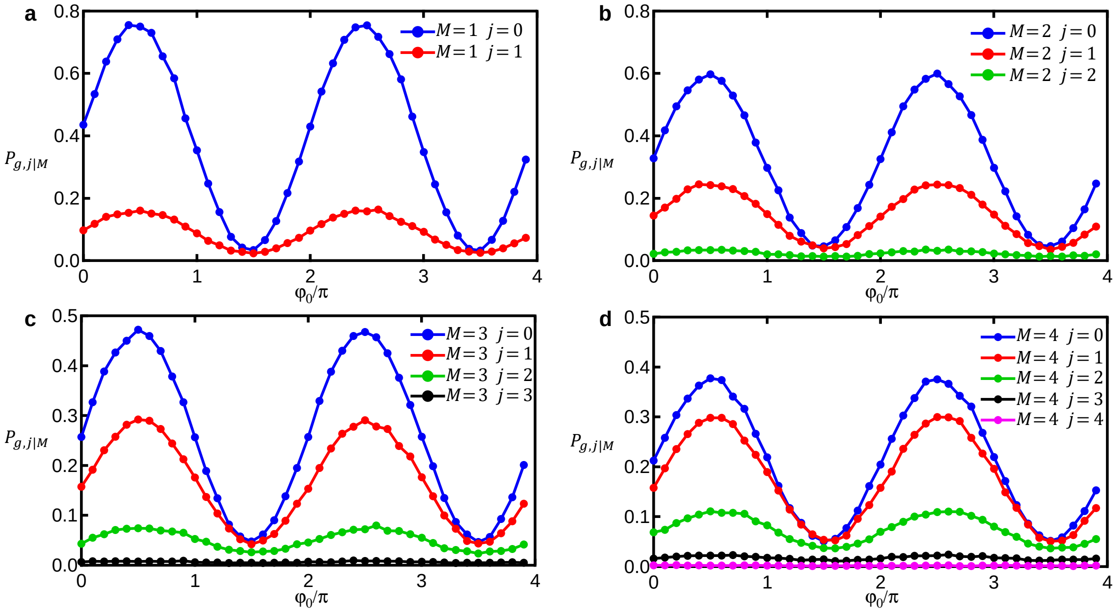

Supplementary Figure 9: **Experimental results in the QJT scheme.** The measured ground state population of the ancilla $P_{\mathrm{g},j|M}$ for the sensing process in which the single-photon-loss error occurs j times with QEC recycled for M times. **a**-**d** are for $M = 1, 2, 3, 4$ respectively. The probe state is $|\psi_{1,3}\rangle = \alpha_{1,3}|1\rangle + \beta_{1,3}|3\rangle$ and each interrogation time is $\tau_{\mathrm{int}} = 0.1T_1$.

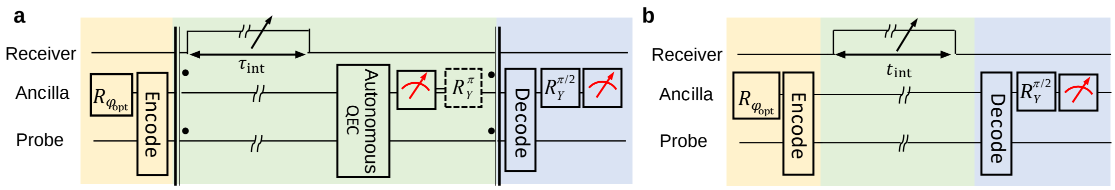

Supplementary Figure 10: **Quantum radiometry scheme. a**, The employed sensing scheme with QEC. The sequence is the same as Fig. 4**a** in the main text except that the probe is prepared at the optimal working point. The sensing signal (an accumulated phase) is converted to the ground state population of the ancilla as a function of the photon number present in the receiver. The QEC process is repeated for M times with each interrogation time τ_{int}, giving the total interaction time $t_{\mathrm{int}} = M\tau_{\mathrm{int}}$. **b**, The sequence for sensing without QEC. For comparison, the interrogation time between the probe and receiver is set close to the total time in the case with QEC.

when implementing the QEC process only once, the sensing signals are demonstrated in Fig. 4**c** of the main text, following the experimental parameters shown in Supplementary Table 2.

Supplementary Fig. 11**c** shows the results with the QJT scheme. The Ramsey fringes show a reduction of the contrast with more occurrence of single-photon-loss errors. From the measured $P_g(p)$, the sensitivity of our radiometry can be calculated, as shown in Fig. 4**d** of the main text.

III. SUPPLEMENTARY NOTE 3 - NUMERICAL ANALYSIS

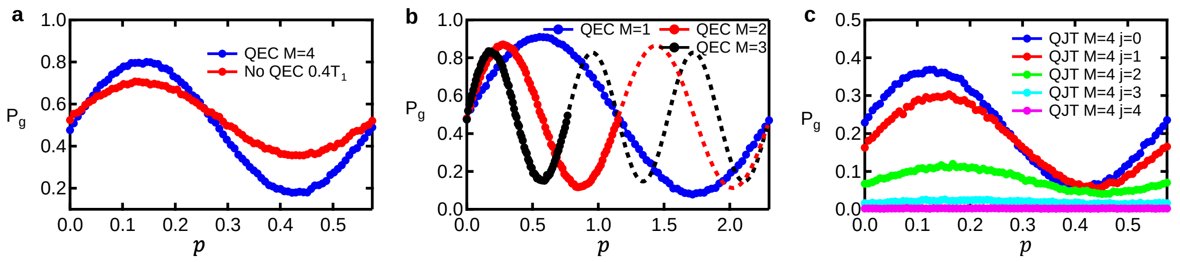

Supplementary Figure 11: **Quantum sensing of photon numbers for $|\psi_{1,3}\rangle$. a**, The comparison of the oscillation fringes between experiments with and without QEC. The red dots represent the ground state population of the ancilla P_g with QEC repeated for $M=4$ times and each interrogation time $\tau_{\rm int}=0.1T_1$, while the blue dots show the results without QEC with the total interrogation time $t_{\rm int}=0.4T_1$. **b**, The measured P_g as a function of the photon number p in the receiver for the probe state $|\psi_{1,3}\rangle$ with different QEC repetitions. The solid dots are experimental data and the dotted lines are plotted from the fitted functions. **c**, The sensing results with the QJT scheme, which are classified in terms of the occurrence numbers of single-photon-loss errors.

A. Model for numerical simulation

Taking the drives for the GRAPE-optimized quantum control into the model, the system evolution (including the initialization, gates, and measurements) could be numerically simulated based on the effective Hamiltonian in the rotating frame as

$$\begin{aligned}H_{\rm eff}/\hbar = & -\chi_{\rm qs}\,|e\rangle\langle e|\,a_{\rm s}^\dagger a_{\rm s}-\chi_{\rm qr}\,|e\rangle\langle e|\,a_{\rm r}^\dagger a_{\rm r}-\chi_{\rm sr}a_{\rm s}^\dagger a_{\rm s}a_{\rm r}^\dagger a_{\rm r}-\frac{K_q}{2}a_{\rm q}^{\dagger 2}a_{\rm q}^2-\frac{K_r}{2}a_{\rm r}^{\dagger 2}a_{\rm r}^2-\frac{K_s}{2}a_s^{\dagger 2}a_s^2-\frac{K_s'}{6}a_s^{\dagger 3}a_s^3\\ & +\Omega_1^{\rm q}(t)(a_{\rm q}^\dagger+a_{\rm q})+i\Omega_2^{\rm q}(t)(a_{\rm q}^\dagger-a_{\rm q})+\Omega_1^{\rm s}(t)(a_{\rm s}^\dagger+a_{\rm s})+i\Omega_2^{\rm s}(t)(a_{\rm s}^\dagger-a_{\rm s})\\ & +\Omega^r(t)(a_{\rm r}^\dagger+a_{\rm r}).\end{aligned}\tag{47}$$

Here, $\Omega_1^{\rm q}(t)$, $\Omega_2^{\rm q}(t)$ and $\Omega_1^{\rm s}(t)$, $\Omega_2^{\rm s}(t)$ are quadratures of the time-dependent drives on the qubit and the probe cavity, respectively. $\Omega^{\rm r}$ is the drive strength for the receiver cavity, mimicking the signal to be detected by the radiometry. The expected value of $\Omega^{\rm r}$ can be calculated by considering the drive power $P_{\rm d}$ applied at frequency $\omega_{\rm r}$ on resonance with the receiver cavity. From the input-output theory, the receiver drive strength is given by $\Omega^r\sim\sqrt{P_{\rm d}}\propto\sqrt{p}\kappa_{\rm r}$, where p is the average photon number in the receiver cavity in the steady state and $\kappa_{\rm r}$ is the decay rate of the receiver cavity. Note that the receiver cavity is a two-sided one with $\kappa_{\rm r}\approx\kappa_{\rm out}\gg\kappa_{\rm in}$, where $\kappa_{\rm out}$ and $\kappa_{\rm in}$ are the output and input coupling constants, respectively. The whole evolution process can be described by the master equation taking into account the relaxations of both the probe and the ancilla qubit. The numerical simulations are implemented by QuTip [15] using the device parameters from the experimental characterization in Supplementary Table 1. The qubit thermal population $n_{\rm th}^{\rm q}\sim0.01$ and the thermal population of the probe $p_{\rm th}^{\rm s}\sim0.01$.

Numerical simulations imitate the experimental protocol and the results are represented in Supplementary Fig. 12, showing good agreement with the experimental results. The deviation of the simulation from the experiment is probably due to high-order interaction terms and decoherence that are not taken into account in the above effective Hamiltonian. Supplementary Fig. 13 shows the simulated photon number sensitivity for different probe states, in good agreement with the experiments.

B. Analysis of imperfections in the real experiment

In Sec. I B, an ideal model is introduced and described, where perfect state initialization, evolution, QEC operation, and detection are assumed, and only the amplitude damping of the probe cavity and the approximate QEC approach to combat this decoherence effect are studied. In practical experiments, imperfections should be taken into consideration, such as the self-Kerr effect, dephasing, and thermal excitations of the probe cavity and the qubit, because they could induce other high-order noise, operation errors, and measurement errors. As shown in Supplementary Fig. 2**b**, the inclusion of these imperfect factors helps to construct a more realistic model. It is worth noting that only the calibrated parameters and the control pulse envelope from GRAPE are used in the simulation, and no further assumptions or parameters are used.

To quantitatively analyze the impact of these imperfections, Fisher information loss (FIL) can be induced:

$$\mathrm{FIL}=\frac{\mathscr{F}_{\rm Ideal}-\mathscr{F}_{\rm Imp}}{\mathscr{F}_{\rm Ideal}},\tag{48}$$

where $\mathscr{F}_{\rm Ideal}$ is the QFI from the ideal model as described above and in Sec. I B, while $\mathscr{F}_{\rm Imp}$ is the QFI when including certain imperfections in the model. The numerical calculation is shown in Supplementary Table 4. It shows that with the increase of n

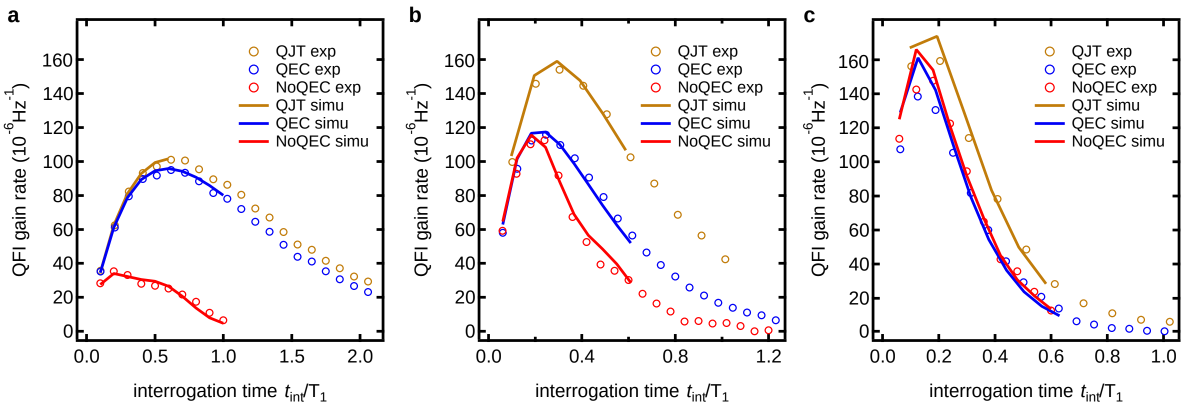

Supplementary Figure 12: **Comparison between experimental and numerical results.** **a**, **b**, and **c**, Simulation results of the QFI gain rate for different probe states $|\psi_{1,3}\rangle$, $|\psi_{1,5}\rangle$, and $|\psi_{1,7}\rangle$, respectively. Lines are the simulated results, in good agreement with dots that represent the experimental data. The optimized coefficients of the probe states and interrogation times are shown in Supplementary Table 2 and Supplementary Table 3. The parameters of the experiment device are shown in Supplementary Table 1.

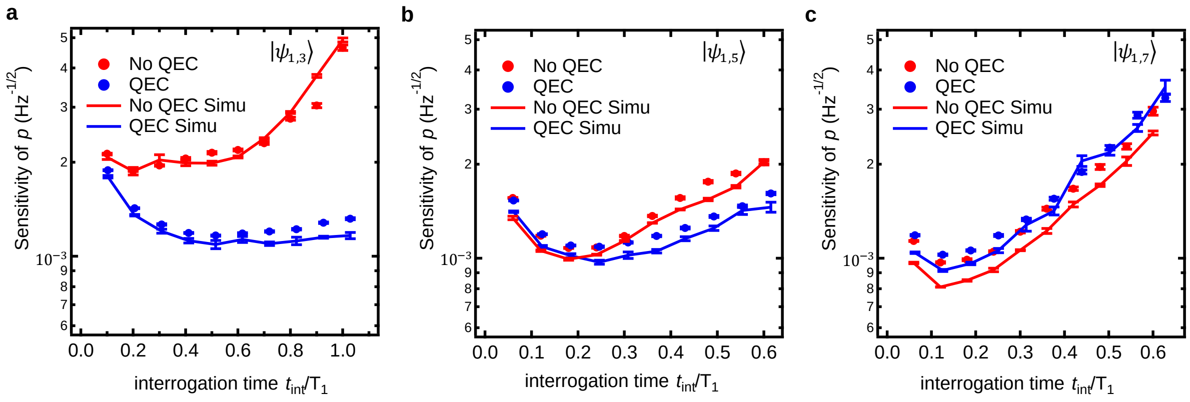

Supplementary Figure 13: **Simulation results of photon number sensitivity for different probe states.** **a**, **b**, and **c**, Results for $|\psi_{1,3}\rangle$, $|\psi_{1,5}\rangle$, and $|\psi_{1,7}\rangle$, respectively. Lines are the simulated results, in good agreement with dots that represent the experimental data. The error bars are obtained through error propagation of the fit parameter uncertainties.

(the higher-level component), the corresponding FIL tends to increase. Among all the imperfections, operation errors during the QEC process have the greatest influence on the sensitivity, followed by the errors during the sensing evolution of the probe and the GRAPE pule error during the measurement process. Errors during the initialization seem to be negligible.

	$\lvert\Psi_{1,3}\rangle$	$\lvert\Psi_{1,5}\rangle$	$\lvert\Psi_{1,7}\rangle$
Initialization	0.02%	0.04%	0.03%
QEC	30.2%	33.3%	37.5%
Evolution	10.7%	12.7%	17.9%
Measurement	5.5%	17.9%	20.2%

Supplementary Table 4: **Fisher information loss (FIL) in different experimental processes**.

In the following, we will investigate in detail the imperfections in the state initialization, evolution, QEC, and detection processes separately and numerically. We note that the parameters of the experimental device is shown in Supplementary Table 1, the optimized coefficients of the initial probe states and the interrogation times are shown in Supplementary Table 2 and Table 3, and the recycle numbers $M = 6,3,2$ for $n = 3,5,7$, respectively, corresponding to the optimal normalized QFI in the real experiment.

1. State initialization imperfection

The initialization process can be divided into two parts. First, the ancilla qubit state is initialized to $|\psi_{m,n}\rangle = \alpha_{m,n}|g\rangle + \beta_{m,n}e^{i\varphi_0}|e\rangle$ with an initial phase (φ_0) and optimized $\alpha_{m,n}$ and $\beta_{m,n}$ (Supplementary Table 2 and Supplementary Table 3) in 20 ns. It is worth noting that higher-level Fock states correspond to higher energies in the probe cavity and experience stronger damping effects, and thus larger n is associated with a higher fidelity loss during the control pulse, which is also the case in the followed processes. Second, the information is encoded from the ancilla qubit to the photonic state in the probe cavity ($|\psi_{m,n}\rangle$) via a GRAPE control pulse with a length of 800 ns. Although the decoherences of the ancilla qubit and the probe mode induce FIL, this process with a short duration only accounts for $0.02\% \sim 0.03\%$ of the FIL.

2. Evolution imperfection

During the sensing evolution process of the probe in a real metrology experiment, the probe quantum state accumulates an extra phase through the cross-Kerr interaction with the receiver cavity. In addition to the amplitude damping error, the probe will also experience the extra self-Kerr effect, thermal excitation, as well as the dephasing due to the ancilla qubit. Via numerical simulations, these imperfections account for $10.7\% \sim 17.9\%$ of the FIL.

3. QEC imperfection

The experimental QEC procedure consists of the following steps.

(i) GRAPE pulse. The QEC is implemented autonomously via a GRAPE pulse (800 ns long) to complete a unitary operation on the composite probe-ancilla system. This autonomous operation could stabilize the probe state to the odd parity: add a photon when the parity changes, i.e. $\left(\alpha_{m,n}\sqrt{m}|m-1\rangle + \beta_{m,n}\sqrt{n}e^{i\theta}|n-1\rangle\right)|g\rangle$ is converted to $\left(\alpha_{m,n}\sqrt{m}|m\rangle + \beta_{m,n}\sqrt{n}e^{i\theta}|n\rangle\right)|e\rangle$ if a single-photon-loss error occurs; perform an identity operation to the probe if the parity is conserved. During this period, there are decoherences in both the probe cavity and the ancilla qubit, as well as imperfections of the GRAPE pulse.

(ii) Qubit measurement. The ancilla qubit state indicates whether the single-photon-loss error has occurred or not. Although the output of the ancilla is not important in conventional QEC process for keeping quantum information, the trajectory of the system evolution is important in our quantum metrology experiments. Therefore, we also both measure and record the ancilla outcomes, and feed the results for the QJT-enhanced sensing. To measure the ancilla qubit, a pulse is injected into the receiver cavity, which will lead to a non-negligible dephasing effect in the probe cavity.

(iii) π-pulse: After the measurement, if an excited state of the ancilla qubit $|e\rangle$ is detected, a π pulse will be carried out with a latency of 336 ns to reset the ancilla qubit to the ground state $|g\rangle$.

For all the processes above, the probe state keeps evolving in the presence of decoherences. Besides, all the mentioned pulses are not perfect, i.e. the fidelity of the gates by the GRAPE-optimized pulses could not be unity even if there is no any decoherence. From our numerical simulations, these factors account for the majority of the FIL about $30.2\% \sim 37.5\%$.

4. Measurement imperfection

In the final measurement process, the amplitude and phase information of the probe is transferred from the probe state back to the ancilla qubit via another 800 ns-long decoding GRAPE pulse. Subsequently, a Hadamard gate and a projection measurement are performed on the ancilla qubit. Similarly, decoherences of the probe and the ancilla during these operations and the imperfect pulses will affect the final interference curve. Additionally, the readout error of the ancilla would also contribute to the measurement imperfection, with the fidelity matrix described in Supplementary Fig. 14. Totally, these two parts contribute to the FIL for about 17.9%.

C. Derivation of the sensitivity

The excitation population of the receiver cavity is derived from the measured phase of the probe state. As shown in the calibration process, the outcomes of the detection show interference curves when varying the virtual phase φ_0 of the probe state $|\Psi_{m,n}\rangle = \alpha_{m,n}|m\rangle + \beta_{m,n}e^{i\varphi_0}|n\rangle$. For example, for the simplest case without QJT, the curves can be fit by $P_{\mathrm{g}}(\omega) = A + B\cos\left[\omega(n-m)t_{\mathrm{int}} + \varphi_0\right]$ and $P_{\mathrm{e}} = 1 - P_{\mathrm{g}}$. Both the accumulated phase of Fock state $|n\rangle$ with respect to $|m\rangle$ and the virtual phase

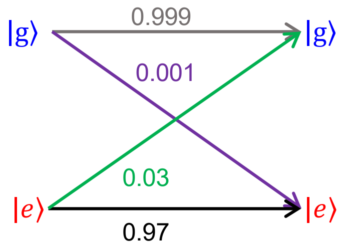

Supplementary Figure 14: **The fidelity matrix of measuring the ancilla qubit state in the experiment.**

induce the interference, and thus the QFI for measuring ω can be calculated as

$$\mathscr{F}=\frac{1}{P_g(\omega)}\left[\frac{\partial P_g(\omega)}{\partial\omega}\right]^2+\frac{1}{P_e(\omega)}\left[\frac{\partial P_e(\omega)}{\partial\omega}\right]^2. \tag{49}$$

The corresponding achievable measurement uncertainty of ω for a single trial of the sensing experiment is

$$\Delta\omega\geq 1/\sqrt{\mathscr{F}}. \tag{50}$$

For a total experimental time t_{tot}, the sensitivity of ω in a unit time reads

$$\sigma_\omega\geq 1/\sqrt{\mathscr{Q}_\omega}, \tag{51}$$

with the normalized QFI

$$\mathscr{Q}_\omega=\mathscr{F}/t_{\mathrm{tot}}. \tag{52}$$

For the radiometry, the cross-Kerr effect induces the frequency shift of the probe, as

$$\delta\omega_{\mathrm{s}}=\chi_{\mathrm{sr}}p, \tag{53}$$

with p being the excitation population in the receiver mode. Therefore, the sensitivity of measuring the excitation population in the receiver mode in the experiment is

$$\sigma_p=\frac{1}{\sqrt{\mathscr{Q}_p}}=\frac{1}{\chi_{\mathrm{sr}}}\sigma_\omega. \tag{54}$$

For different experimental procedures, $\mathscr{Q}_\omega$ or $\mathscr{Q}_p$ are derived from experimental data, and more details could be found in the Methods section of the main text. Especially, the measurement outputs are grouped according to the numbers of detected quantum jumps and separately processed.

We now provide the detail of the data analysis in Fig. 3 and Fig. 4 of the main text at the optimal point with the probe state $|\psi_{1,5}\rangle$ (Fig. 3**c**, Fig. 4**e**). In Fig. 3**c** of the main text, the normalized QFI for the QEC+QJT strategy is calculated as

$$\begin{aligned}\mathscr{Q}_\omega=&\frac{1}{t_{\mathrm{tot}}}\sum_{j=0}^{M=3}\sum_{l\in\{g,e\}}\frac{B_{l,j}^2(n-m)^2t_{\mathrm{int}}^2}{A_{l,j}}\\=&\frac{(5-1)^2(14500\,\mathrm{ns}\times 3)^2}{(14500\,\mathrm{ns}+1484\,\mathrm{ns})\times 3+1940\,\mathrm{ns}}\times 0.2538\\=&1.54\times 10^{-4}\,\mathrm{Hz}^{-1},\end{aligned} \tag{55}$$

where t_{tot} includes the interrogation time $\tau_{\mathrm{int}}=14500\,\mathrm{ns}$, QEC operation time of 1484 ns (both are repeated for three times), as well as the duration of 1940 ns for encoding and decoding. For the results in Fig. 4**e** of the main text, we have

$$\begin{aligned}\mathscr{Q}_p=&\frac{1}{t_{\mathrm{tot}}}\sum_{j=0}^{M}\sum_{l\in\{g,e\}}\frac{1}{P_{l,j|M}}\left[\frac{\partial P_{l,j|M}}{\partial p}\right]^2\\=&\frac{1}{(14500\,\mathrm{ns}+1484\,\mathrm{ns})\times 3+1940\,\mathrm{ns}}\times 60.52\\=&1.21\times 10^{6}\,\mathrm{Hz}.\end{aligned} \tag{56}$$

Here, $\frac{\partial P_{l,j|M}}{\partial p}$ is obtained directly from the slope of experimental curve against p. Therefore, we obtain the sensitivity

$$\begin{aligned}\sigma_p =&\frac{1}{\sqrt{\mathscr{Q}_p}}\\ =&9.1\times10^{-4}\,\mathrm{Hz}^{-1/2}.\end{aligned} \tag{57}$$

[1] S. L. Braunstein and C. M. Caves, "Statistical distance and the geometry of quantum states," Phys. Rev. Lett. **72**, 3439 (1994).

[2] S. Zhou, M. Zhang, J. Preskill, and L. Jiang, "Achieving the Heisenberg limit in quantum metrology using quantum error correction," Nat. Commun. **9**, 78 (2018).

[3] H. K. Ng and P. Mandayam, "Simple approach to approximate quantum error correction based on the transpose channel," Phys. Rev. A **81**, 062342 (2010).

[4] M. A. Nielsen and I. L. Chuang, *Quantum Computation and Quantum Information* (Cambridge Univ. Press, 2000).

[5] L. Sun, A. Petrenko, Z. Leghtas, B. Vlastakis, G. Kirchmair, K. M. Sliwa, A. Narla, M. Hatridge, S. Shankar, J. Blumoff, L. Frunzio, M. Mirrahimi, M. H. Devoret, and R. J. Schoelkopf, "Tracking photon jumps with repeated quantum non-demolition parity measurements," Nature **511**, 444 (2014).

[6] H. M. Wiseman and G. J. Milburn, *Quantum measurement and control* (CAMBRIDGE UNIVERSITY PRESS, 2014) pp. 231–249.

[7] A. Fujiwara, "Quantum channel identification problem," Phys. Rev. A **63**, 042304 (2001).

[8] S. E. S. Nigg, H. Paik, B. Vlastakis, G. Kirchmair, S. Shankar, L. Frunzio, M. H. Devoret, R. J. Schoelkopf, and S. M. Girvin, "Black-Box Superconducting Circuit Quantization," Phys. Rev. Lett. **108**, 240502 (2012).

[9] W. Wang, Y. Wu, Y. Ma, W. Cai, L. Hu, X. Mu, Y. Xu, Z.-J. Chen, H. Wang, Y. P. Song, H. Yuan, C.-L. Zou, L.-M. Duan, and L. Sun, "Heisenberg-limited single-mode quantum metrology in a superconducting circuit," Nat. Commun. **10**, 4382 (2019).

[10] S. Krastanov, V. V. Albert, C. Shen, C.-L. Zou, R. W. Heeres, B. Vlastakis, R. J. Schoelkopf, and L. Jiang, "Universal control of an oscillator with dispersive coupling to a qubit," Phys.Rev.A **92**, 040303 (2015).

[11] N. Khaneja, T. Reiss, C. Kehlet, T. Schulte-Herbrüggen, and S. J. Glaser, "Optimal control of coupled spin dynamics: design of nmr pulse sequences by gradient ascent algorithms," J. Magn. Reson. **172**, 296 (2005).

[12] P. De Fouquieres, S. Schirmer, S. Glaser, and I. Kuprov, "Second order gradient ascent pulse engineering," J. Magn. Reson. **212**, 412 (2011).

[13] L. Hu, Y. Ma, W. Cai, X. Mu, Y. Xu, W. Wang, Y. Wu, H. Wang, Y. Song, C. Zou, S. M. Girvin, L.-M. Duan, and L. Sun, "Quantum error correction and universal gate set on a binomial bosonic logical qubit," Nat. Phys. **15**, 503 (2019).

[14] J. Gambetta, A. Blais, D. I. Schuster, A. Wallraff, L. Frunzio, J. Majer, M. H. Devoret, S. M. Girvin, and R. J. Schoelkopf, "Qubit-photon interactions in a cavity: Measurement-induced dephasing and number splitting," Phys. Rev. A **74**, 042318 (2006).

[15] J. Johansson, P. Nation, and F. Nori, "Qutip: An open-source python framework for the dynamics of open quantum systems," Comp. Phys. Comm. **183**, 1760 (2012).
