# Supplementary material for: Quantum-enhanced radiometry via approximate quantum error correction
Source: Nat Commun. 2022 Jun 9;13:3214. doi: 10.1038/s41467-022-30410-8 (PMC9184621; doi:10.1038/s41467-022-30410-8)
Supplement: Supplementary file 2 — Description of Additional Supplementary Information [file 41467_2022_30410_MOESM2_ESM.pdf]

Description of Additional Supplementary Files

1. **File Name:** Data_Fig2c

   Description: Source data of Figure2 c in the main text.

2. **File Name:** Data_Fig2d

   Description: Source data of Figure2 d in the main text.

3. **File Name:** Data_Fig3b

   Description: Source data of Figure3 b in the main text.

4. **File Name:** Data_Fig3c

   Description: Source data of Figure3 c in the main text.

5. **File Name:** Data_Fig3d

   Description: Source data of Figure3 d in the main text.

6. **File Name:** Data_Fig4b

   Description: Source data of Figure4 b in the main text.

7. **File Name:** Data_Fig4c

   Description: Source data of Figure4 c in the main text.

8. **File Name:** Data_Fig4d

   Description: Source data of Figure4 d in the main text.

9. **File Name:** Data_Fig4e

   Description: Source data of Figure4 e in the main text.

10. **File Name:** Data_Fig4f

    Description: Source data of Figure4 f in the main text.

Note that the data above are provided in the spreadsheets of “Supplementary Dataset.xlsx”.
